# Securing the sensing functionality in ISAC: KLD-based ambiguity function shaping

**DOI:** 10.1038/s44459-026-00089-0

**Published:** 2026-10-01

**Authors:** Borui Du, Kawon Han, Christos Masouros

**Affiliations:** 1 Dept. of Electronic and Electrical Engineering, University College London, London, UK; 2 Dept. of Electrical Engineering, Ulsan National Institute of Science and Technology, Ulsan, South Korea

**Keywords:** Engineering, Mathematics and computing, Physics

## Abstract

Integrated sensing and communication (ISAC) systems introduce a sensing-privacy vulnerability because unauthorized sensing eavesdroppers (Eve) may exploit ISAC signals for independent passive sensing. This work addresses sensing-security for orthogonal frequency-division multiplexing (OFDM)- and orthogonal time frequency space (OTFS)-based ISAC waveforms from a target-detection perspective. We develop system models for the base station (BS), user equipment, and Eve, and define a Kullback-Leibler divergence-based detection metric that accounts for mainlobe, sidelobe, and noise components in ambiguity-function and range-Doppler maps. Building on this analysis, we formulate a sensing-secure signaling design problem that tunes a perturbation matrix to jointly control signal amplitude and phase in the time-frequency domain and solve it via simulated annealing. Simulation results show that the proposed scheme substantially degrades Eve’s detection probability—from 79.4% to 37.4% for OTFS and from 94.3% to 33.0% for OFDM—while incurring only a small loss in BS sensing performance. The proposed scheme also enables controllable sensing-security and communication trade-offs.

## Introduction

With the rapid expansion of wireless technology, the coverage of wireless devices is increasing significantly, and managing the expanding population of devices and signal types is becoming increasingly costly in terms of spectrum and physical infrastructure. From the perspective of the technology paradigm, the two main functions of radio-communication and sensing are combinable; therefore, integrated sensing and communication (ISAC) is regarded as a significant direction for future wireless technology development^1,2^. Communication uses a radio signal to convey information from the source, through the channel, to the sink, while sensing uses a radio signal to capture information from the source of the channel to the sink-both follow the same transmitter-channel-receiver process. Moreover, to eliminate channel-introduced distortion, communication performs channel estimation and equalization, and channel estimation essentially captures information from the channel as a sensing function, indicating strong potential for combining the two functions in demand and technological essentials.

Given that ISAC uses the same signal to convey information and sense the environment, new security vulnerabilities arise for the ISAC system, which have become a significant concern in recent studies^3–5^. This concern is treated as an ISAC security problem with two facets: data security, which protects the confidentiality of communication information during sensing^4,6,7^, and sensing-security, which protects the sensing targets from unauthorized sensing^3,8,9^. The sensing-security threat in ISAC is particularly critical because an unauthorized receiver, referred to as the sensing eavesdropper (Eve), can exploit the base station (BS)-emitted ISAC signal for passive sensing without transmitting any probing waveform. This silent and non-cooperative operation makes Eve extremely difficult to expose, detect, or localize, allowing it to acquire sensitive environmental information without the awareness of the legitimate BS. Such information may include traffic flows, object locations, aerial targets, and other critical physical-world dynamics. Moreover, since ISAC waveforms are intentionally designed for high sensing accuracy, Eve may obtain precise spatio-temporal descriptions of the surrounding environment. Therefore, sensing-security is essential for preventing the unintended use of ISAC signals for unauthorized passive sensing. Although attention to ISAC security has grown in recent years, most efforts address data security, leaving sensing-security comparatively underdeveloped. Advancing sensing-secured ISAC is therefore increasingly important as the other side of the coin in future ISAC security.

Early efforts focused on data security, assuming that a sensing target acts as communication Eve and attempts to intercept the information-bearing component of the ISAC signal. Su et al.^10,11^ propose two physical-layer data security schemes for multiple-antenna ISAC systems: one minimizes the signal-to-noise ratio (SNR) at Eve while constraining legitimate communication and sensing, and the other exploits destructive interference to deteriorate Eve’s eavesdropping. Afterwards, Su et al.^6^ propose a two-stage design that first estimates the target angles and then maximizes the secrecy rate and optimizes the Cramér-Rao bound (CRB) through joint beamforming and artificial-noise (AN) covariance design. Liu et al.^7^ extend AN-aided protection to orthogonal time frequency space (OTFS) waveforms. They show secrecy gains with or without knowledge of the eavesdropper’s channel state information (CSI) by adapting beamforming or injecting AN into the null space.

Before ISAC sensing-security emerged as a distinct topic, sensing privacy had been studied in legacy wireless systems, especially Wi-Fi. Early CSI- or signal-obfuscation methods protect Wi-Fi localization or sensing privacy by randomizing preambles/pilots, injecting phase noise, or perturbing the physical information carried by communication signals^12–16^. For example, PhyCloak^14^ obfuscates sensing information embedded in communication signals, WiCloak^15^ injects fake channel responses to protect Wi-Fi device location privacy while maintaining normal communication, and Secur-Fi^16^ generates recoverable fraud signals to block unauthorized Wi-Fi sensing while supporting authorized sensing. Other works exploit multi-input multi-output (MIMO) capabilities for sensing privacy protection. MIMO-based schemes use phase-difference injection, neural-network-aided recovery, or physical-layer channel encryption to protect sensing privacy while enabling authorized CSI reconstruction^17–19^; for instance, MIMOCrypt^19^ exploits MIMO to physically encrypt Wi-Fi sensing channels for multi-user privacy-preserving sensing. In addition, external-device-assisted approaches have also been investigated, including intelligent reconfigurable surface (IRS)-based channel obfuscation, metasurface-enabled sensing attacks, and backscatter-tag-assisted defenses^20–22^. For example, IRShield^20^ uses an intelligent reflecting surface to obfuscate wireless channels against adversarial sensing, and ScatterShield^22^ employs backscatter tags to interfere with CSI features used by unauthorized Wi-Fi sensing. These studies provide important insights into wireless sensing privacy and security, but their protection mechanisms still have several limitations, including dependence on CSI-based sensing, reliance on key-based or coding-like encryption/recovery, limited physical interpretability in black-box designs, and the need for additional external devices. Therefore, physically interpretable sensing-security mechanisms that directly exploit waveform-domain sensing properties for ISAC systems remain worth further investigation.

Recent work has therefore broadened the security focus from data eavesdropping to sensing-security. Guo et al.^23^ discuss countermeasures such as cooperative jamming, directional antennas, and beamforming. Qu et al.^3^ survey privacy and security in ISAC and formalize attacker models that eavesdrop on motion parameters or jam reflections. In general, the sensing-security solutions can be divided into two types: Eve-aware and Eve-agnostic^24^. The sensing-security problem has been widely investigated in an Eve-aware paradigm, where the BS can leverage some prior information about sensing eavesdroppers. Zou et al.^8^ propose AN-aided beamforming that protects the sensing function, where a communication user is taken as the sensing eavesdropper, and the design maximizes the mutual information (MI) of legitimate sensing while constraining that of the eavesdropper. Based on an active Eve model, Li et al.^25^ study an Eve-aware non-orthogonal multiple access-based ISAC system that balances achievable communication rates with CRB-based sensing constraints through joint subcarrier scheduling, beamforming, and jamming.

Note that the above solutions typically build on the availability of Eve’s locations and channels at the legitimate ISAC BS. In some scenarios, however, a sensing Eve may remain silent and thus may not readily reveal their presence, making it difficult to acquire accurate channel knowledge of such eavesdroppers. Consequently, recent research has also explored Eve-agnostic sensing-security in ISAC systems. Wang et al.^26^ introduce a diffusion-model-based safeguarding signal for Eve-agnostic sensing-security in orthogonal frequency-division multiplexing (OFDM) ISAC systems and report superior performance over existing baselines. Very recently, Han et al.^9^ explicitly exploit ambiguity function (AF) shaping for OFDM to enhance Eve-agnostic sensing-security by controlling subcarrier power to insert artificial peaks into the range-Doppler (RD) response of Eve’s sensing. Chen et al.^27^ design an Eve-agnostic sensing-security scheme for near-field ISAC systems that exploits environmental scatterers to deceive sensing eavesdroppers.

Nevertheless, most existing works remain largely focused on OFDM waveforms and theory-oriented metrics such as MI and CRB for sensing-security design in ISAC systems, and a unified framework with practical sensing-security metrics and multi-waveform support is still unexplored. Other waveform candidates, such as frequency-modulated continuous-wave (FMCW), OTFS, and affine frequency-division multiplexing (AFDM), exhibit distinct sensing-communication trade-offs that call for dedicated sensing-security designs^28–32^. From an AF perspective, these waveform choices induce different RD structures and sensing-security leakage patterns, providing a unifying lens that links waveform structure to resolvability and unintended sensing. Recent AF-based studies on single-carrier, OFDM, and OTFS clarify how waveform structure governs the sensing-communication trade-off^33,34^, while for AFDM, a closed-form AF expression has been derived and parameter regimes with reduced sidelobes have been identified^35^.

Although existing sensing privacy protection methods provide useful insights, their dependence on CSI-based processing, key-based recovery, black-box models, or external devices limits their direct applicability to physically interpretable waveform-level sensing-security design in ISAC systems. In addition, current waveform and AF analyses do not adequately account for sensing-security performance, leaving the interaction among waveform design, AF behavior, and sensing-security insufficiently understood. For current ISAC sensing-security studies, two limitations dominate: many studies rely on theoretical metrics and models without validation on practical waveforms, and dependence on AN imposes a power burden that benefits only security. Consequently, ISAC sensing-security remains early-stage, with threat models and evaluation metrics still maturing. In such conditions, this study investigates the sensing-security problem from a detection perspective, aiming to secure the sensing functionality in ISAC systems. The proposed approach is applicable to both Eve-aware and Eve-agnostic cases, thereby bridging the two regimes. Our contributions are summarized as follows:

- We develop system models for user equipment (UE) communication, legitimate BS sensing, and Eve’s passive sensing under OFDM and OTFS waveform operations. Building on the system models, we decompose the received sensing RD response at both the BS and Eve into mainlobe, sidelobe, and noise components guided by Wiener filtering, or so-called linear minimum mean square error (LMMSE) filtering, and matched filtering (MF) receiver properties. This yields closed-form expressions for the effective sensing-signal-to-interference-plus-noise ratio (SINR) contributions for detection under OFDM and OTFS waveform operations. The decomposition links these components to detection performance for legitimate and unauthorized sensing.
- We define a Kullback-Leibler divergence (KLD) metric between the sensing distributions for the target-absent and target-present cases at both the BS and Eve. To enable efficient optimization, we apply Jensen’s inequality to the expectation terms and derive a tight, computable surrogate of the derived KLD. This surrogate retains the KLD’s dependence on the waveform design and is therefore suitable as a tractable objective for subsequent sensing-security optimization. Based on the derived KLD, we define the KLD gap between the legitimate BS and Eve as a sensing-security metric.
- Using the KLD-based metrics together with a communication-error penalty, we formulate a sensing-security optimization over a perturbation matrix that controls amplitude and phase in the time-frequency (TF) domain under standard power constraints. In this optimization, Eve’s KLD is expressed in terms of Eve’s channel coefficients and noise power, allowing for an Eve-aware design. In practice, however, these quantities can be set to nominal Eve parameters rather than Eve’s exact parameters, so that the method can be used in Eve-agnostic scenarios. We solve the resulting nonconvex problem using simulated annealing (SA).

Our Monte Carlo simulations show significant gains: the proposed design consistently elevates Eve’s sidelobes and reduces their detection probability while keeping the BS’s sensing degradation small. By varying the priority weight between sensing-security and communication, the proposed method demonstrates security improvements and characterizes the sensing-security-communication trade-off. It reveals that stronger sensing-security introduces a graceful degradation of communication errors and the data rate, while lowering Eve’s detection probability, thereby enhancing resilience to unauthorized sensing.

The remainder of this paper is organized as follows. The Methods section presents the system model, including the scenario model and its assumptions, followed by the ISAC signal model for BS, UE, and Eve. It also presents the mainlobe, sidelobe, and noise component decomposition of the RD response, the KLD-based secure sensing analysis for both BS and Eve, and the optimization problem solved using the SA algorithm. The Results section presents the simulation results and interpretation. Finally, the Discussion section concludes the paper and outlines future directions.

In this paper, matrices and vectors are denoted by bold letters, e.g., **A** and **a** or $\underline{a}$, while ${\left[{\bf{A}}\right]}_{i,j}$ represents the element in the *i*-th row and *j*-th column of matrix **A**. ${\mathbb{C}}$ and ${\mathbb{R}}$ represent the complex and real number sets, respectively. The operators (⋅)^*T*^, (⋅)^*^, and (⋅)^*H*^ denote matrix transpose, conjugate, and conjugate transpose, respectively. The notation ${\mathcal{CN}}(0,{\bf{C}})$ represents a zero-mean circularly symmetric complex Gaussian (CSCG) distribution with covariance matrix **C**. ⊙ and ⊘ are element-wise product and division. The trace, vectorization, and Kronecker product operators are denoted as ${\rm{Tr}}(\cdot )$, vec(⋅), and ⊗, respectively. vec^−1^(⋅) is the reshape function that transforms a vector into a matrix, and ${\rm{diag}}\left(\cdot \right)$ is the diagonal matrix operator. Expectation is represented by ${\mathbb{E}}\{\cdot \}$. The normalized discrete Fourier matrix and the identity matrix of dimension *N* are denoted as **F**_*N*_ and **I**_*N*_, respectively. ∣⋅∣, ∥⋅∥_2_ and ∥⋅∥_*F*_ are element-wise absolute value, *l*_2_ norm, and Frobenius norm.

## Methods

**Fig. 1:**
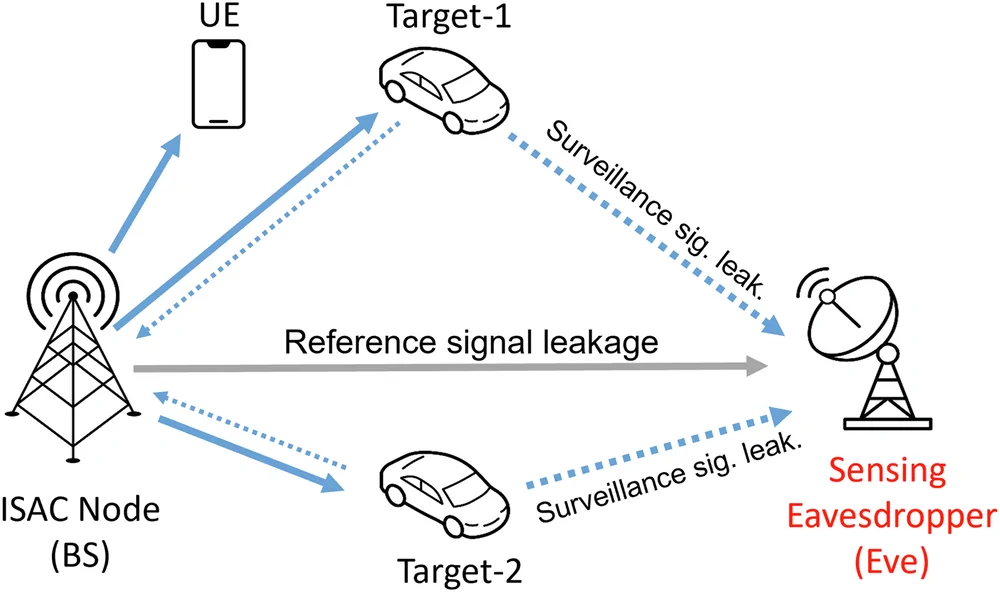
Scenario model of the considered ISAC sensing-security system. The figure illustrates a base station (BS), user equipment (UE), multiple sensing targets, and a passive sensing eavesdropper (Eve). The BS transmits an integrated sensing and communication (ISAC) waveform for simultaneous UE communication and environmental sensing, while Eve attempts passive sensing by exploiting the BS-emitted signal.

In this section, we first specify the system model and its underlying assumptions. The ISAC system executes joint sensing and communications via a single BS. We focus on a specific scenario that contains one UE, multiple targets, and one passive sensing eavesdropper. The BS radiates a unified ISAC waveform through a single-input single-output antenna that simultaneously: (i) delivers data to the UE and (ii) probes the environment to detect the existence of targets, in a given RD bin. Eve remains silent and attempts to detect the same targets through passive sensing using the BS waveform, where the direct-path signal serves as the reference, and the reflected-path signal serves as the surveillance observation. The illustration of the scenario model is presented in Fig. 1.

To facilitate the analysis and focus on the essential property of the sensing-security scenario, we impose some assumptions for the system^9^. Particularly, we develop a signaling design that is applicable to both Eve-aware and Eve-agnostic scenarios.

- **A.1:** Depending on the scenario, the BS may have or lack knowledge of Eve-related parameters (e.g., target-channel coefficients and noise power), covering both Eve-aware and Eve-agnostic settings.
- **A.2:** Eve knows the BS’s location, synchronization parameters, and carrier frequency, but is unaware of the exact transmitted signal structure, where the transmitted signal carries random data and no pilot symbols.
- **A.3:** Eve has no prior information about the target.
- **A.4:** Eve possesses sufficient spatial resources (e.g., antenna elements) to extract a reference signal with enough separation from their direct channel to the BS.
- **A.5:** The target moves slowly compared to the resolution of the transmitted signal. Therefore, the sensing function can be performed using TF domain processing with the approximation in ref. ^36^.

Related to **A.2**, since pilots are known reference signals, Eve may exploit them for pilot-assisted or CSI-based sensing. In this work, we focus on data-bearing passive sensing, where the transmitted signal consists of random data symbols only, so as to formulate a KLD-based sensing-security framework for unauthorized sensing from data-bearing signals. Under **A.4**, Eve is assumed to have passive processing capabilities, including reference/surveillance separation and direct-path interference cancellation, to suppress the strong direct BS–Eve component before extracting weak target-reflected signals^37,38^.

### Transmitted signal model

The transmitted OFDM and OTFS signals can be represented as a general form in the TF domain with perturbation, which is expressed as

1 $${{\bf{x}}}_{{\rm{TF}}}={{\bf{W}}}_{{\rm{TF}}}{{\bf{s}}}_{{\rm{TF}}}={{\bf{W}}}_{{\rm{TF}}}{\bf{U}}{\bf{s}},$$

where ${{\bf{s}}}_{{\rm{TF}}}\in {{\mathbb{C}}}^{MN}$ is the data symbol in the TF domain, and ${\bf{U}}\in {{\mathbb{C}}}^{MN\times MN}$ is the transform matrix for different modulation methods. *M* is the number of subcarriers, and *N* is the number of time-slots. For OFDM, **U** = **I**_*MN*_, indicating that the communication symbol coincides with the TF samples. For OTFS, ${\bf{U}}=\left({{\bf{F}}}_{N}^{H}\otimes {{\bf{F}}}_{M}\right)$, which represents the inverse symplectic finite Fourier transform (ISFFT) to map the delay-Doppler (DD) domain symbols into the TF domain. Note that **U** is a unitary matrix for both OFDM and OTFS, and typically for other waveforms. Throughout this paper, we model the data vector ${\bf{s}}\in {{\mathbb{C}}}^{MN}$ as having independent, identically distributed elements with zero mean and unit variance but with specific constellations, i.e., ${\mathbb{E}}\{{\bf{s}}\}={\bf{0}}$ and ${\mathbb{E}}\{{\bf{s}}{{\bf{s}}}^{H}\}={{\bf{I}}}_{MN}$. ${{\bf{W}}}_{{\rm{TF}}}\in {{\mathbb{C}}}^{MN\times MN}$ denotes the complex perturbation matrix in the TF domain, enabling both amplitude and phase control; specifically, ${{\bf{W}}}_{{\rm{TF}}}={\rm{diag}}({[{w}_{1},\ldots ,{w}_{MN}]}^{T})$, where each ${w}_{i}\in {\mathbb{C}}$ applies an independent per TF-bin perturbation, assuming

2 $$\begin{array}{r}{\rm{Tr}}\left({{\bf{W}}}_{\,\mathrm{TF}\,}^{H}{{\bf{W}}}_{{\rm{TF}}}\right)=\mathop{\sum }\limits_{i=1}^{MN}| {w}_{i}{| }^{2}=MN.\end{array}$$

Consequently, the total energy of the modulated signal is *MN*, while the power for each symbol is controlled by **W**_TF_. Then, the TF domain signal, **x**_TF_, is transformed using inverse fast Fourier transform for transmission, which can be expressed as follows

3 $$\begin{array}{r}{{\bf{x}}}_{{\rm{t}}}=\left({{\bf{I}}}_{N}\otimes {{\bf{F}}}_{M}^{H}\right){{\bf{x}}}_{{\rm{TF}}},\end{array}$$

where ${{\bf{x}}}_{{\rm{t}}}\in {{\mathbb{C}}}^{MN}$ is the transmitted signal in the time domain.

### Communication signal model

Using the Rician model, which contains line-of-sight (LoS) and non-line-of-sight (NLoS) components, the communication channel between the BS and UE can be expressed as follows

4 $${{\bf{H}}}_{{\rm{c}}}={\alpha }_{c}\sqrt{\frac{{\kappa }_{c}}{{\kappa }_{c}+1}}\,{{\mathbf{\Pi }}}_{MN}^{{l}_{c}}{{\mathbf{\Delta }}}_{MN}^{{k}_{c}}+{\alpha }_{c}\sqrt{\frac{1}{{\kappa }_{c}+1}}{{\bf{H}}}_{{\rm{c,NLoS}}},$$

where ${{\bf{H}}}_{{\rm{c}}}\in {{\mathbb{C}}}^{MN\times MN}$ and *α*_*c*_, *κ*_*c*_, *l*_*c*_, and *k*_*c*_ are the path-loss, Rician factor, delay, and Doppler shift of the communication channel, respectively. In this work, we assume integer delay and Doppler for brevity, and ${l}_{s,p}^{\,\mathrm{BS}\,}$, ${k}_{s,p}^{\,\mathrm{BS}\,}$, ${l}_{s,p}^{\,\mathrm{E}\,}$, and ${k}_{s,p}^{\,\mathrm{E}\,}$ are normalized by the corresponding resolutions in the simulation setting^39^. The addition and removal of the cyclic-prefix (CP) are assumed to be performed before parameter estimation. Therefore, the delay operation in the delay domain is modeled as a circular shift, i.e., ${{\mathbf{\Pi }}}_{MN}^{{l}_{c}}$ and ${{\mathbf{\Delta }}}_{MN}^{{k}_{c}}$ are delay shift and Doppler phase matrices as shown in^39^. Besides, the elements of **H**_c,NLoS_ follow ${\mathcal{CN}}\left(0,1\right)$, which represents the NLoS channel in the transmission.

The received signal in the symbol multiplexing domain for the UE can be formulated as

5 $$\begin{array}{l}{{\bf{y}}}_{\mathrm{sym}}=\mathop{\underbrace{{{\bf{U}}}^{H}({{\bf{I}}}_{N}\otimes {{\bf{F}}}_{M}){{\bf{H}}}_{{\rm{c}}}({{\bf{I}}}_{N}\otimes {{\bf{F}}}_{M}^{H})}}\limits_{\triangleq {{\bf{H}}}_{{\rm{c}},\mathrm{eff}}}{{\bf{W}}}_{\mathrm{TF}}{\bf{U}}{\bf{s}}\\\qquad\,\;+\mathop{\underbrace{{{\bf{U}}}^{H}({{\bf{I}}}_{N}\otimes {{\bf{F}}}_{M}){{\bf{z}}}_{{\rm{c}}}}}\limits_{\triangleq {{\bf{z}}}_{{\rm{c}},\mathrm{eff}}}\end{array}$$

6 $$={{\bf{H}}}_{{\rm{c,eff}}}{{\bf{W}}}_{{\rm{TF}}}{\bf{U}}{\bf{s}}+{{\bf{z}}}_{{\rm{c,eff}}},$$

where ${{\bf{z}}}_{{\rm{c,eff}}} \sim {\mathcal{CN}}\left({\bf{0}},{\sigma }_{{{\bf{z}}}_{{\rm{c}}}}^{2}{{\bf{I}}}_{MN}\right)$ is the effective additive white Gaussian noise (AWGN) after the inverse unitary transform of ${{\bf{U}}}^{H}\left({{\bf{I}}}_{N}\otimes {{\bf{F}}}_{M}\right)$, and the ${\sigma }_{{{\bf{z}}}_{{\rm{c}}}}^{2}$ is the variance of the noise for the communication function. Therefore, the effective sequence-level rate metric for a specific data sequence is given by

7 $${R}_{{\rm{c}}}=\log \left[1+\frac{{\left\Vert {\bf{s}}\right\Vert }_{2}^{2}}{{\left\Vert {{\bf{H}}}_{\mathrm{c},\mathrm{eff}}{{\bf{W}}}_{{\rm{TF}}}{\bf{U}}{\bf{s}}-{\bf{s}}\right\Vert }_{2}^{2}+{\left\Vert {{\bf{z}}}_{\mathrm{c},\mathrm{eff}}\right\Vert }_{2}^{2}}\right],$$

where ${\left\Vert {{\bf{H}}}_{\mathrm{c},\mathrm{eff}}{{\bf{W}}}_{{\rm{TF}}}{\bf{U}}{\bf{s}}-{\bf{s}}\right\Vert }_{2}^{2}$ is the mismatch component between the effective data sequence after the communication channel and the desired data sequence at the UE side^40,41^. The form of Eq. (7) is used as a sequence-level communication metric to quantify the distortion introduced by the channel and the proposed TF-domain perturbation. This allows the sensing-secured ISAC design to focus on the waveform-level trade-off between communication performance and sensing-security protection, rather than on a particular equalization or decoding architecture. Consequently, for fixed data-sequence energy and noise power, minimizing the mismatch component based on the channel information of **H**_c,eff_ is equivalent to maximizing this effective rate metric. In a more general communication design, **W**_TF_ can be extended from an element-wise TF perturbation matrix to a linear precoding or pre-equalization matrix to compensate for communication-channel distortion while preserving the sensing-security functionality.

### Legitimate monostatic sensing signal model

The BS performs sensing in a monostatic mode. Based on the LoS channel model and assuming the velocity is small (**A.5**), we have the following expression of the sensing channel representation in the TF domain

8 $${{\bf{H}}}_{{\rm{BS,s}}}=\mathop{\sum }\limits_{p=1}^{K}{\alpha }_{s,p}^{\mathrm{BS}\,}\cdot {\underline{\psi }}^{H}\left({l}_{s,p}^{\,\mathrm{BS}\,}\right)\underline{\phi }\left({k}_{s,p}^{\,\mathrm{BS}\,}\right),$$

9 $${\mathrm{with}}\,\underline{\psi }\left({l}_{s,p}^{\,{\mathrm{BS}}\,}\right)=\exp \left(\left[0,j2\pi \frac{{l}_{s,p}^{\,{\mathrm{BS}}\,}}{M},\cdots \,,j2\pi \frac{{l}_{s,p}^{\,{\mathrm{BS}}\,}(M-1)}{M}\right]\right),$$

10 $$\,{\mathrm{and}}\,\underline{\phi }\left({k}_{s,p}^{\,{\mathrm{BS}}\,}\right)=\exp \left(\left[0,{j}2\pi \frac{{k}_{s,p}^{\,{\mathrm{BS}}\,}}{N},\cdots \,,j2\pi \frac{{k}_{s,p}^{\,{\mathrm{BS}}\,}(N-1)}{N}\right]\right),$$

where ${{\bf{H}}}_{{\rm{BS,s}}}\in {{\mathbb{C}}}^{M\times N}$ and *K* is the number of targets in the scenario. The coefficient ${\alpha }_{s,p}^{\,\mathrm{BS}\,}$ corresponds to the deterministic path-loss for target-*p*, including radar cross section. Additionally, ${l}_{s,p}^{\,\mathrm{BS}\,}$ and ${k}_{s,p}^{\,\mathrm{BS}\,}$ are round-trip delay and Doppler shifts, respectively. $\underline{\psi }$ and $\underline{\phi }$ are the delay and Doppler phase vectors. Thus, the received sensing signal of BS can be written as

11 $${{\bf{R}}}_{{\rm{BS,s}}}={{\bf{H}}}_{{\rm{BS,s}}}\odot {{\bf{X}}}_{{\rm{TF}}}+{{\bf{Z}}}_{{\rm{BS,s,TF}}},$$

where ${{\bf{R}}}_{{\rm{BS,s}}}\in {{\mathbb{C}}}^{M\times N}$, and ${{\bf{X}}}_{{\rm{TF}}}\triangleq {{\rm{vec}}}^{-1}\left({{\bf{x}}}_{{\rm{TF}}}\right)$ represents the samples in the TF domain. ${{\bf{Z}}}_{{\rm{BS,s,TF}}}\triangleq {{\rm{vec}}}^{-1}\left[\left({{\bf{I}}}_{N}\otimes {{\bf{F}}}_{M}\right){{\bf{z}}}_{{\rm{BS,s}}}\right]$ is the noise transformed into the TF domain. ${{\bf{z}}}_{{\rm{BS,s}}} \sim {\mathcal{CN}}\left(0,{\sigma }_{{{\bf{z}}}_{{\rm{BS,s}}}}^{2}{{\bf{I}}}_{MN}\right)$, and ${\sigma }_{{{\bf{z}}}_{{\rm{BS,s}}}}^{2}$ is defined as the noise variance for BS’s sensing functionality.

### Eve sensing signal model

The Eve performs passive sensing in a bistatic mode. Similar to the LoS sensing channel of the BS, the sensing target for Eve can be represented as follows:

12 $${{\bf{H}}}_{{\rm{E,s}}}=\mathop{\sum }\limits_{p=1}^{K}{\alpha }_{s,p}^{\mathrm{E}\,}\cdot {\underline{\psi }}^{H}\left({l}_{s,p}^{\,\mathrm{E}\,}\right)\underline{\phi }\left({k}_{s,p}^{\,\mathrm{E}\,}\right),$$

where ${{\bf{H}}}_{{\rm{E,s}}}\in {{\mathbb{C}}}^{M\times N}$ and ${\alpha }_{s,p}^{\,\mathrm{E}\,}$, ${l}_{s,p}^{\,\mathrm{E}\,}$ and ${k}_{s,p}^{\,\mathrm{E}\,}$ are different from ${\alpha }_{s,p}^{\,\mathrm{BS}\,}$, ${l}_{s,p}^{\,\mathrm{BS}\,}$, and ${k}_{s,p}^{\,\mathrm{BS}\,}$ given the different geometry between BS’s and Eve’s perspectives. The received signal model of Eve can be represented as

13 $${{\bf{R}}}_{{\rm{E,s}}}={{\bf{H}}}_{{\rm{E,s}}}\odot {{\bf{X}}}_{{\rm{TF}}}+{{\bf{Z}}}_{{\rm{E,s,TF}}},$$

where ${{\bf{R}}}_{{\rm{E,s}}}\in {{\mathbb{C}}}^{M\times N}$ and **Z**_E,s,TF_ is the noise in the TF domain for Eve. ${{\bf{z}}}_{{\rm{E,s}}} \sim {\mathcal{CN}}\left(0,{\sigma }_{{{\bf{z}}}_{{\rm{E,s}}}}^{2}{{\bf{I}}}_{MN}\right)$, and ${\sigma }_{{{\bf{z}}}_{{\rm{E,s}}}}^{2}$ is defined as the noise variance for Eve’s sensing functionality.

Because Eve does not know the transmitted signal from the BS, they need to extract the reference signal from the direct channel, which is assumed to be a Rician channel and can be represented as

14 $${{\bf{H}}}_{{\rm{E,d}}}={\alpha }_{d}^{\mathrm{E}\,}\sqrt{\frac{{\kappa }_{e}}{{\kappa }_{e}+1}}{{\bf{H}}}_{{\rm{E,d,LoS}}}+{\alpha }_{d}^{\mathrm{E}\,}\sqrt{\frac{1}{{\kappa }_{e}+1}}{{\bf{H}}}_{{\rm{E,d,NLoS}}},$$

where ${{\bf{H}}}_{{\rm{E,d}}}\in {{\mathbb{C}}}^{M\times N}$ and ${\alpha }_{d}^{\,\mathrm{E}\,}$ is the path-loss of the direct channel between BS and Eve, and *κ*_*e*_ is the Rician factor, and

15 $${{\bf{H}}}_{{\rm{E,d,LoS}}}={\underline{\psi }}^{H}\left({l}_{d}^{\,\mathrm{E}\,}\right)\underline{\phi }\left({k}_{d}^{\,\mathrm{E}\,}\right),$$

where ${{\bf{H}}}_{{\rm{E,d,LoS}}}\in {{\mathbb{C}}}^{M\times N}$ and ${l}_{d}^{\,\mathrm{E}\,}$ and ${k}_{d}^{\,\mathrm{E}\,}$ are the motion parameters for the direct channel of Eve. The elements of **H**_E,d,NLoS_ follow ${\mathcal{CN}}\left(0,1\right)$. Therefore, the received signal from the direct channel has the following form

16 $${{\bf{R}}}_{{\rm{E,d}}}={{\bf{H}}}_{{\rm{E,d}}}\odot {{\bf{X}}}_{{\rm{TF}}}+{{\bf{Z}}}_{{\rm{E,d,TF}}},$$

where ${{\bf{R}}}_{{\rm{E,d}}}\in {{\mathbb{C}}}^{M\times N}$ and **Z**_E,d,TF_ is the noise in the TF domain for Eve’s direct link. ${{\bf{z}}}_{{\rm{E,d}}} \sim {\mathcal{CN}}\left(0,{\sigma }_{{{\bf{z}}}_{{\rm{E,d}}}}^{2}{{\bf{I}}}_{MN}\right)$, and ${\sigma }_{{{\bf{z}}}_{{\rm{E,d}}}}^{2}$ is defined as the noise variance for Eve’s direct link towards the BS.

Since Eve is assumed to infer the deterministic LoS component **H**_E,d,LoS_ from the known BS location and synchronization information, it can compensate the LoS DD effect in the direct-path observation by element-wise division. Specifically, from Eq. (16), Eve obtains

17 $$\begin{array}{ll}{\widehat{{\bf{X}}}}_{{\rm{E,d,TF}}}\triangleq {{\bf{R}}}_{{\rm{E,d}}}\oslash {{\bf{H}}}_{{\rm{E,d,LoS}}}\\ \qquad\quad\;\;={\alpha }_{d}^{\,\mathrm{E}\,}\sqrt{\frac{{\kappa }_{e}}{{\kappa }_{e}+1}}{{\bf{X}}}_{{\rm{TF}}}+{\alpha }_{d}^{\mathrm{E}\,}\sqrt{\frac{1}{{\kappa }_{e}+1}}{\widetilde{{\bf{H}}}}_{{\rm{E,d}}}\odot {{\bf{X}}}_{{\rm{TF}}}\\ \qquad\qquad+{\widetilde{{\bf{Z}}}}_{{\rm{E,d,TF}}},\end{array}$$

where ⊘ denotes element-wise division, ${\widetilde{{\bf{H}}}}_{{\rm{E,d}}}\triangleq {{\bf{H}}}_{{\rm{E,d,NLoS}}}\oslash {{\bf{H}}}_{{\rm{E,d,LoS}}}$, and ${\widetilde{{\bf{Z}}}}_{{\rm{E,d,TF}}}\triangleq {{\bf{Z}}}_{{\rm{E,d,TF}}}\oslash {{\bf{H}}}_{{\rm{E,d,LoS}}}$. Therefore, ${\widehat{{\bf{X}}}}_{{\rm{E,d,TF}}}$ serves as Eve’s reference observation for passive sensing, which contains the transmitted TF-domain signal together with residual NLoS distortion and compensated noise.

This section presents the sensing receiver processing for the BS and Eve, then decomposes the contributions of the mainlobe, sidelobes, and noise on their respective sensing performance. Building on this analysis, we develop a KLD-based analysis of detection performance at the BS and Eve, defining the sensing-security metric as the KLD gap between the legitimate BS and Eve for secure-sensing signaling design.

### BS sensing receiver processing

Since the legitimate BS has full knowledge of the transmitted signal **X**_TF_, the target parameters can be estimated using an LMMSE filter. The LMMSE filter is known to outperform the reciprocal filter (RF) and MF by controlling sidelobes given the signal and noise statistics^42^, as follows:

18 $${\left[{{\bf{G}}}_{{\rm{BS}}}\right]}_{m,n}=\frac{{x}_{m,n}^{* }}{{\left\vert {x}_{m,n}\right\vert }^{2}+{\sigma }_{{{\bf{z}}}_{{\rm{BS,s}}}}^{2}}=\frac{{w}_{m,n}^{* }{s}_{m,n}^{* }}{{\left\vert {w}_{m,n}{s}_{m,n}\right\vert }^{2}+{\sigma }_{{{\bf{z}}}_{{\rm{BS,s}}}}^{2}},$$

where ${x}_{m,n}={\left[{{\bf{X}}}_{{\rm{TF}}}\right]}_{m,n}={w}_{m,n}{s}_{m,n}$. *w*_*m*,*n*_ ≜ *w*_*nM*+*m*_, ${s}_{m,n}\triangleq {\left[{{\bf{S}}}_{{\rm{TF}}}\right]}_{m,n}$, and ${{\bf{S}}}_{{\rm{TF}}}\in {{\mathbb{C}}}^{M\times N}\triangleq {{\rm{vec}}}^{-1}\left({{\bf{s}}}_{{\rm{TF}}}\right)$. The filtered signal in the TF domain has the following form

19 $${\left[{\widehat{{\bf{H}}}}_{{\rm{BS,s}}}\right]}_{m,n}={\left[{{\bf{G}}}_{{\rm{BS}}}\right]}_{m,n}\cdot {\left[{{\bf{R}}}_{{\rm{BS,s}}}\right]}_{m,n}$$

20 $$\begin{array}{ll}&=\frac{{\left\vert {x}_{m,n}\right\vert }^{2}}{{\left\vert {x}_{m,n}\right\vert }^{2}+{\sigma }_{{{\bf{z}}}_{{\rm{BS,s}}}}^{2}}{\left[{{\bf{H}}}_{{\rm{BS,s}}}\right]}_{m,n}\\ &+\frac{{x}_{m,n}^{* }}{{\left\vert {x}_{m,n}\right\vert }^{2}+{\sigma }_{{{\bf{z}}}_{{\rm{BS,s}}}}^{2}}{\left[{{\bf{Z}}}_{{\rm{BS,s,TF}}}\right]}_{m,n},\end{array}$$

where ${\widehat{{\bf{H}}}}_{{\rm{BS,s}}}\in {{\mathbb{C}}}^{M\times N}$ is the estimated channel matrix in the TF domain. Using the symplectic finite Fourier transform, the estimated channel will be transformed into the DD domain, i.e., RD map, as follows

21 $${\widehat{{\mathbf{\Lambda }}}}_{{\rm{BS}}}={{\bf{F}}}_{M}^{H}{\widehat{{\bf{H}}}}_{{\rm{BS,s}}}{{\bf{F}}}_{N},$$

which can be detailed as

22 $${\left[{\widehat{{\mathbf{\Lambda }}}}_{{\rm{BS}}}\right]}_{l,k}=\frac{1}{\sqrt{MN}}\sum _{m,n}{\left[{\widehat{{\bf{H}}}}_{{\rm{BS,s}}}\right]}_{m,n}{e}^{j2\pi \left(\frac{ml}{M}-\frac{nk}{N}\right)},$$

where *l* = 0, 1,⋯, *M* − 1 and *k* = 0, 1,⋯, *N* − 1. Therefore, the estimate ${\widehat{{\mathbf{\Lambda }}}}_{{\rm{BS}}}$ can be decomposed into target channel component and noise components as below

23 $$\begin{array}{ll}{\left[{\widehat{{\mathbf{\Lambda }}}}_{{\rm{BS}}}\right]}_{l,k}&=\frac{1}{\sqrt{MN}}\mathop\sum \limits_{m,n}\frac{{\left\vert {x}_{m,n}\right\vert }^{2}}{{\left\vert {x}_{m,n}\right\vert }^{2}+{\sigma }_{{{\bf{z}}}_{{\rm{BS,s}}}}^{2}}{\left[{{\bf{H}}}_{{\rm{BS,s}}}\right]}_{m,n}\\ &\times \exp \left\{\,j2\pi \left(\frac{m\,l}{M}-\frac{n\,k}{N}\right)\right\}\\ &+\frac{1}{\sqrt{MN}}\mathop\sum\limits _{m,n}\frac{{x}_{m,n}^{* }}{{\left\vert {x}_{m,n}\right\vert }^{2}+{\sigma }_{{{\bf{z}}}_{{\rm{BS,s}}}}^{2}}{\left[{{\bf{Z}}}_{{\rm{BS,s,TF}}}\right]}_{m,n}\\ &\times \exp \left\{\,j2\pi \left(\frac{m\,l}{M}-\frac{n\,k}{N}\right)\right\}\\ \end{array}$$

24 $$\triangleq {\left[{\widehat{{\mathbf{\Lambda }}}}_{{\bf{H}}}^{{\rm{BS}}}\right]}_{l,k}+{\left[{\widehat{{\mathbf{\Lambda }}}}_{{\bf{Z}}}^{{\rm{BS}}}\right]}_{l,k}.$$

Given the target-*p* is located at $({l}_{s,p}^{\,\mathrm{BS}},{k}_{s,p}^{\mathrm{BS}\,})$, the channel component, ${\left[{\widehat{{\mathbf{\Lambda }}}}_{{\bf{H}}}^{{\rm{BS}}}\right]}_{{l}_{s,p}^{{\rm{BS}}},{k}_{s,p}^{{\rm{BS}}}}$, of the estimate RD map at $({l}_{s,p}^{\,\mathrm{BS}},{k}_{s,p}^{\mathrm{BS}\,})$ can be simplified as

25 $$\begin{array}{l}{\left[{\widehat{{\mathbf{\Lambda }}}}_{{\bf{H}}}^{{\rm{BS}}}\right]}_{{l}_{s,p}^{{\rm{BS}}},{k}_{s,p}^{{\rm{BS}}}}=\frac{1}{MN}\mathop\sum\limits _{m,n}\frac{{\left\vert {x}_{m,n}\right\vert }^{2}}{{\left\vert {x}_{m,n}\right\vert }^{2}+{\sigma }_{{{\bf{z}}}_{{\rm{BS,s}}}}^{2}}\mathop\sum\limits _{l,k}{[{{\mathbf{\Lambda }}}_{{\rm{BS,s}}}]}_{l,k}\\ \times \exp \left\{j2\pi \,\left(\frac{m\,({l}_{s,p}^{\,\mathrm{BS}\,}-l)}{M}+\frac{n\,(k-{k}_{s,p}^{\,\mathrm{BS}\,})}{N}\right)\right\},\end{array}$$

26 $$\begin{array}{ll}&=\frac{1}{MN}\mathop\sum\limits _{m,n}\frac{| {x}_{m,n}{| }^{2}}{| {x}_{m,n}{| }^{2}+{\sigma }_{{{\bf{z}}}_{{\rm{BS,s}}}}^{2}}{\alpha }_{s,p}^{\mathrm{BS}\,}\\ &+\frac{1}{MN}\mathop{\sum }\limits_{\begin{array}{l}q=1\\ q\ne p\end{array}}^{K}\mathop\sum\limits _{m,n}\frac{| {x}_{m,n}{| }^{2}}{| {x}_{m,n}{| }^{2}+{\sigma }_{{{\bf{z}}}_{{\rm{BS,s}}}}^{2}}{\alpha }_{s,q}^{\mathrm{BS}\,}\\ &\times \exp \left\{j2\pi \,\left(\frac{m\,({l}_{s,p}^{\,\mathrm{BS}}-{l}_{s,q}^{\mathrm{BS}\,})}{M}+\frac{n\,({k}_{s,q}^{\,\mathrm{BS}}-{k}_{s,p}^{\mathrm{BS}\,})}{N}\right)\right\},\end{array}$$

where **Λ**_BS,s_ is the actual target in the RD map from the BS’s perspective. We further define the mainlobe and sidelobe interference component from other targets in Eq. (26) as follows

27 $${\left[{\widehat{{\mathbf{\Lambda }}}}_{{\bf{S}}}^{{\rm{BS}}}\right]}_{{l}_{s,p}^{{\rm{BS}}},{k}_{s,p}^{{\rm{BS}}}}\triangleq \frac{1}{MN}\sum _{m,n}\frac{| {x}_{m,n}{| }^{2}}{| {x}_{m,n}{| }^{2}+{\sigma }_{{{\bf{z}}}_{{\rm{BS,s}}}}^{2}}{\alpha }_{s,p}^{\mathrm{BS}\,},$$

28 $${\left[{\widehat{{\mathbf{\Lambda }}}}_{{\bf{I}}}^{{\rm{BS}}}\right]}_{{l}_{s,p}^{{\rm{BS}}},{k}_{s,p}^{{\rm{BS}}}}\triangleq \frac{1}{\sqrt{MN}}\mathop{\sum }\limits_{\begin{array}{c}q=1\\ q\ne p\end{array}}^{K}{\alpha }_{s,q}^{\mathrm{BS}\,}{\left[{\widetilde{{\mathbf{\Lambda }}}}_{{\rm{BS}}}\right]}_{({l}_{s,p}^{{\rm{BS}}}-{l}_{s,q}^{{\rm{BS}}}),({k}_{s,p}^{{\rm{BS}}}-{k}_{s,q}^{{\rm{BS}}})},$$

where ${\widetilde{{\mathbf{\Lambda }}}}_{{\rm{BS}}}$ denotes the RD response of the LMMSE filter for detection at the BS side when the desired target is located at ${l}_{s}^{\,\mathrm{BS}\,}=0$ and ${k}_{s}^{\,\mathrm{BS}\,}=0$. Therefore,

29 $${\left[{\widehat{{\mathbf{\Lambda }}}}_{{\bf{H}}}^{{\rm{BS}}}\right]}_{{l}_{s,p}^{{\rm{BS}}},{k}_{s,p}^{{\rm{BS}}}}={\left[{\widehat{{\mathbf{\Lambda }}}}_{{\bf{S}}}^{{\rm{BS}}}\right]}_{{l}_{s,p}^{{\rm{BS}}},{k}_{s,p}^{{\rm{BS}}}}+{\left[{\widehat{{\mathbf{\Lambda }}}}_{{\bf{I}}}^{{\rm{BS}}}\right]}_{{l}_{s,p}^{{\rm{BS}}},{k}_{s,p}^{{\rm{BS}}}}.$$

Besides, the noise component can be written as

30 $$\begin{array}{ll}{\left[{\widehat{{\mathbf{\Lambda }}}}_{{\bf{Z}}}^{{\rm{BS}}}\right]}_{l,k}&=\frac{1}{\sqrt{MN}}\mathop\sum\limits _{m,n}\frac{{x}_{m,n}^{* }}{{\left\vert {x}_{m,n}\right\vert }^{2}+{\sigma }_{{{\bf{z}}}_{{\rm{BS,s}}}}^{2}}{\left[{{\bf{Z}}}_{{\rm{BS,s,TF}}}\right]}_{m,n}\\ &\times \exp \left[\,j2\pi \,\left(\frac{m\,l}{M}-\frac{n\,k}{N}\right)\right],\end{array}$$

where *l* = 0, 1,⋯, *M* − 1 and *k* = 0, 1,⋯, *N* − 1. Therefore, the mainlobe component contains the desired information associated with target-*p* and can be used to detect the target and estimate its parameters, while the other components act as sidelobe interference.

Given the existence of a target at *l*_*t*_ and *k*_*t*_ range and Doppler bin, the detection SINR can be defined as follows:

31 $$\,\mathrm{SINR}\,=\frac{{\mathbb{E}}\left\{{\left\vert {\left[{\widehat{{\mathbf{\Lambda }}}}_{{\bf{S}}}\right]}_{{l}_{t},{k}_{t}}\right\vert }^{2}\right\}}{{\mathbb{E}}\left\{{\left\vert {\left[{\widehat{{\mathbf{\Lambda }}}}_{{\bf{I}}}\right]}_{{l}_{t},{k}_{t}}\right\vert }^{2}\right\}+{\mathbb{E}}\left\{{\left\vert {\left[{\widehat{{\mathbf{\Lambda }}}}_{{\bf{Z}}}\right]}_{{l}_{t},{k}_{t}}\right\vert }^{2}\right\}}.$$

To make this ratio explicit, we next detail the signal and noise powers for the BS and Eve.

Given a specific sequence of data transmission, the numerator, i.e., the expected norm-squared mainlobe for the estimated RD map, has the following expression for the BS:

32 $${P}_{s,p,\mathrm{BS}}\triangleq {\mathbb{E}}\left\{{\left| {\left[{\widehat{{\mathbf{\Lambda }}}}_{{\bf{S}}}^{{\rm{BS}}}\right]}_{{l}_{s,p}^{{\rm{BS}}},{k}_{s,p}^{{\rm{BS}}}}\right| }^{2}\right\}$$

33 $$=\frac{{\left\vert {\alpha }_{s,p}^{{\rm{BS}}}\right\vert }^{2}}{{\left(MN\right)}^{2}}{\left| \sum _{m,n}\frac{| {x}_{m,n}{| }^{2}}{| {x}_{m,n}{| }^{2}+{\sigma }_{{{\bf{z}}}_{{\rm{BS,s}}}}^{2}}\right| }^{2},$$

where $${x}_{m,n}\triangleq {\left[{{\bf{X}}}_{{\rm{TF}}}\right]}_{m,n}$$, and

34 $${\mu }_{p,\mathrm{BS}}\triangleq \frac{{\alpha }_{s,p}^{\mathrm{BS}\,}}{MN}\sum _{m,n}\frac{| {x}_{m,n}{| }^{2}}{| {x}_{m,n}{| }^{2}+{\sigma }_{{{\bf{z}}}_{{\rm{BS,s}}}}^{2}}.$$

Having established the signal terms, we now quantify the associated noise contributions. The noise power for the BS is

35 $${P}_{n,\mathrm{BS}}\triangleq {\mathbb{E}}\left\{{\left| {\left[{\widehat{{\mathbf{\Lambda }}}}_{{\bf{Z}}}^{{\rm{BS}}}\right]}_{{l}_{s,p}^{{\rm{BS}}},{k}_{s,p}^{{\rm{BS}}}}\right| }^{2}\right\}$$

36 $$=\frac{{\sigma }_{{{\bf{z}}}_{{\rm{BS,s}}}}^{2}}{MN}\sum _{m,n}\frac{| {x}_{m,n}{| }^{2}}{{\left(| {x}_{m,n}{| }^{2}+{\sigma }_{{{\bf{z}}}_{{\rm{BS,s}}}}^{2}\right)}^{2}}.$$

The interference produced by other targets at the cell of target-*p* can be expressed as

37 $${\left[{\widehat{{\mathbf{\Lambda }}}}_{{\bf{I}}}^{{\rm{BS}}}\right]}_{{l}_{s,p}^{{\rm{BS}}},{k}_{s,p}^{{\rm{BS}}}}=\frac{1}{\sqrt{MN}}\mathop{\sum }\limits_{\begin{array}{c}q=1\\ q\ne p\end{array}}^{K}{\alpha }_{s,q}^{\mathrm{BS}\,}{\left[{\widetilde{{\mathbf{\Lambda }}}}_{{\rm{BS}}}\right]}_{({l}_{s,p}^{{\rm{BS}}}-{l}_{s,q}^{{\rm{BS}}}),({k}_{s,p}^{{\rm{BS}}}-{k}_{s,q}^{{\rm{BS}}})}.$$

Based on the sidelobe-interference approximation justified in Supplementary Note 1, the aggregate sidelobe interference is modeled as a zero-mean CSCG variable because it is the sum of independently sampled sidelobe contributions with different phase rotations from different interfering targets. Its variance is characterized by the integrated sidelobe level (ISL), ISL_BS_. Therefore, the cross-terms vanish and the interference power is obtained as

38 $${P}_{i,p,\mathrm{BS}}\triangleq {\mathbb{E}}\left\{{\left| {\left[{\widehat{{\mathbf{\Lambda }}}}_{{\bf{I}}}^{{\rm{BS}}}\right]}_{{l}_{s,p}^{{\rm{BS}}},{k}_{s,p}^{{\rm{BS}}}}\right| }^{2}\right\}=\frac{1}{MN}\mathop{\sum }\limits_{\begin{array}{c}q=1\\ q\ne p\end{array}}^{K}{\left\vert {\alpha }_{s,q}^{{\rm{BS}}}\right\vert }^{2}{\mathrm{ISL}}_{{\rm{BS}}},$$

where the target-specific reflection information is contained in ${\alpha }_{s,q}^{\,\mathrm{BS}\,}$ for all interfering targets, and ISL_BS_ is formulated as

39 $${\mathrm{ISL}}_{{\rm{BS}}}=\frac{1}{MN}\left(\sum _{l,k}{\left| {\widetilde{{\mathbf{\Lambda }}}}_{{\rm{BS}}}[l,k]\right| }^{2}-{\left| {\widetilde{{\mathbf{\Lambda }}}}_{{\rm{BS}}}[0,0]\right| }^{2}\right)$$

40 $$=\frac{1}{MN}\left(\sum _{m,n}{\gamma }_{m,n}^{2}-\frac{1}{MN}{\left| \sum _{m,n}{\gamma }_{m,n}\right| }^{2}\right),$$

where ${\gamma }_{m,n}\triangleq \frac{| {x}_{m,n}{| }^{2}}{| {x}_{m,n}{| }^{2}+{\sigma }_{{{\bf{z}}}_{\mathrm{BS},s}}^{2}}$, and the second equality holds because of the energy preservation property of the Fourier transform.

### Eve sensing receiver processing

Consistent with the passive sensing literature and according to **A.5**, the direct path from the BS to the receiver is extracted as a reference, and classical radar processing is performed on the surveillance channel^43–46^. Accordingly, Eve filters the TF signal from the surveillance channel, and we assume they use an MF as the receive filter. The MF maximizes the output SNR and requires no knowledge of the CP or frame structure, remaining effective even when the reference-signal SNR is degraded^9^.

By contrast, alternative mismatched filters generally demand detailed signal-format information, direct-link noise statistics, or direct-link LoS characteristics that are unavailable to Eve^9^. For both optimal output SNR and implementation simplicity, we therefore assume that Eve adopts the MF, which in the TF domain is given by

41 $${\left[{\widehat{{\bf{H}}}}_{{\rm{E,s}}}\right]}_{m,n}={\left[{{\bf{G}}}_{{\rm{E}}}\right]}_{m,n}\cdot {\left[{{\bf{R}}}_{{\rm{E,s}}}\right]}_{m,n},$$

where ${\left[{{\bf{G}}}_{{\rm{E}}}\right]}_{m,n}={x}_{m,n}^{* }$. Observe that **G**_E_ is the original transmitted signal, as known at the BS, in order to facilitate the analysis, which models Eve’s best-case processing (and therefore the BS’s worst-case). In realistic settings, imperfections in Eve’s reference signal introduce performance loss, consequently degrading their detection performance^9^.

Similar to the BS, ${\widehat{{\bf{H}}}}_{{\rm{E,s}}}$ needs to be transformed into the DD domain to have an RD map for target detection, which can be written as

42 $${\widehat{{\mathbf{\Lambda }}}}_{{\rm{E}}}={{\bf{F}}}_{M}^{H}{\widehat{{\bf{H}}}}_{{\rm{E,s}}}{{\bf{F}}}_{N}.$$

Further,

43 $${\left[{\widehat{{\mathbf{\Lambda }}}}_{{\rm{E}}}\right]}_{l,k}=\frac{1}{\sqrt{MN}}\sum _{m,n}{\left[{\widehat{{\bf{H}}}}_{\mathrm{E},s}\right]}_{m,n}{e}^{j2\pi \left(\frac{ml}{M}-\frac{nk}{N}\right)},$$

where *l* = 0, 1,⋯, *M* − 1 and *k* = 0,1,⋯,*N* − 1. Similarly, we define

44 $${\left[{\widehat{{\mathbf{\Lambda }}}}_{{\bf{S}}}^{{\rm{E}}}\right]}_{{l}_{s,p}^{{\rm{E}}},{k}_{s,p}^{{\rm{E}}}}\triangleq \frac{1}{MN}\sum _{m,n}{\left\vert {x}_{m,n}\right\vert }^{2}{\alpha }_{s,p}^{\,\mathrm{E}\,},$$

45 $${\left[{\widehat{{\mathbf{\Lambda }}}}_{{\bf{I}}}^{{\rm{E}}}\right]}_{{l}_{s,p}^{{\rm{E}}},{k}_{s,p}^{{\rm{E}}}}\triangleq \frac{1}{\sqrt{MN}}\mathop{\sum }\limits_{\begin{array}{c}q=1\\ q\ne p\end{array}}^{K}{\alpha }_{s,q}^{\mathrm{E}\,}{\left[{\widetilde{{\mathbf{\Lambda }}}}_{{\rm{E}}}\right]}_{({l}_{s,p}^{{\rm{E}}}-{l}_{s,q}^{{\rm{E}}}),({k}_{s,p}^{{\rm{E}}}-{k}_{s,q}^{{\rm{E}}})},$$

where ${\widetilde{{\mathbf{\Lambda }}}}_{{\rm{E}}}$ is the AF as Eve applies the MF. The noise component follows

46 $${\left[{\widehat{{\mathbf{\Lambda }}}}_{{\bf{Z}}}^{{\rm{E}}}\right]}_{l,k}=\frac{1}{\sqrt{MN}}\sum _{m,n}{x}_{m,n}^{* }{\left[{{\bf{Z}}}_{{\rm{E,s,TF}}}\right]}_{m,n}{e}^{j2\pi \left(\frac{ml}{M}-\frac{nk}{N}\right)},$$

where *l* = 0, 1,⋯, *M* − 1 and *k* = 0, 1,⋯, *N* − 1. Similar to the BS, Eve can utilize the mainlobe component to detect and estimate the parameters of target-*p*.

As in the component decomposition in the previous subsection, from Eq. (31) we present the corresponding expressions for the eavesdropper. For Eve, the mainlobe of the desired target-*p* follows:

47 $${P}_{s,p,\mathrm{E}}\triangleq {\mathbb{E}}\left\{{\left| {\left[{\widehat{{\mathbf{\Lambda }}}}_{{\bf{S}}}^{{\rm{E}}}\right]}_{{l}_{s,p}^{{\rm{E}}},{k}_{s,p}^{{\rm{E}}}}\right| }^{2}\right\}=\frac{{\left\vert {\alpha }_{s,p}^{{\rm{E}}}\right\vert }^{2}}{{\left(MN\right)}^{2}}{\left| \sum _{m,n}| {x}_{m,n}{| }^{2}\right|}^{2},$$

and ${\mu }_{p,\mathrm{E}}\triangleq \frac{{\alpha }_{s,p}^{\mathrm{E}\,}}{MN}\sum _{m,n}| {x}_{m,n}{| }^{2}$. The amplified noise power for Eve follows

48 $${P}_{n,\mathrm{E}}={\mathbb{E}}\left\{{\left| {\left[{\widehat{{\mathbf{\Lambda }}}}_{{\bf{Z}}}^{{\rm{E}}}\right]}_{{l}_{s,p}^{{\rm{E}}},{k}_{s,p}^{{\rm{E}}}}\right| }^{2}\right\}=\frac{{\sigma }_{{{\bf{z}}}_{{\rm{E,s}}}}^{2}}{MN}\sum _{m,n}| {x}_{m,n}{| }^{2}.$$

Similar to the BS, the interference produced by other targets at Eve for the cell of target-*p* can be expressed as

49 $${\left[{\widehat{{\mathbf{\Lambda }}}}_{{\bf{I}}}^{{\rm{E}}}\right]}_{{l}_{s,p}^{{\rm{E}}},{k}_{s,p}^{{\rm{E}}}}=\frac{1}{\sqrt{MN}}\mathop{\sum }\limits_{\begin{array}{c}q=1\\ q\ne p\end{array}}^{K}{\alpha }_{s,q}^{\mathrm{E}\,}{\left[{\widetilde{{\mathbf{\Lambda }}}}_{{\rm{E}}}\right]}_{({l}_{s,p}^{{\rm{E}}}-{l}_{s,q}^{{\rm{E}}}),({k}_{s,p}^{{\rm{E}}}-{k}_{s,q}^{{\rm{E}}})}.$$

Similar to the BS-side, the interference power is obtained as

50 $${P}_{i,p,\mathrm{E}}\triangleq {\mathbb{E}}\left\{{\left\vert {\left[{\widehat{{\mathbf{\Lambda }}}}_{{\bf{I}}}^{{\rm{E}}}\right]}_{{l}_{s,p}^{{\rm{E}}},{k}_{s,p}^{{\rm{E}}}}\right\vert }^{2}\right\}=\frac{1}{MN}\mathop{\sum }\limits_{\begin{array}{c}q=1\\ q\ne p\end{array}}^{K}{\left\vert {\alpha }_{s,q}^{{\rm{E}}}\right\vert }^{2}{\mathrm{ISL}}_{{\rm{E}}},$$

where the target-specific reflection information is contained in ${\alpha }_{s,q}^{\,\mathrm{E}\,}$ for all interfering targets, and the ISL is expressed as

51 $${\mathrm{ISL}}_{{\rm{E}}}=\frac{1}{MN}\left[\sum _{\begin{array}{c}l,k\end{array}}{\left| {\left[{\widetilde{{\mathbf{\Lambda }}}}_{{\rm{E}}}\right]}_{l,k}\right| }^{2}-{\left| {\left[{\widetilde{{\mathbf{\Lambda }}}}_{{\rm{E}}}\right]}_{0,0}\right| }^{2}\right]$$

52 $$=\frac{1}{MN}\left[\sum _{\begin{array}{c}m,n\end{array}}{\left| | {x}_{m,n}{| }^{2}\right| }^{2}-\frac{1}{MN}{\left| \sum _{\begin{array}{c}m,n\end{array}}{\left\vert {x}_{m,n}\right\vert }^{2}\right| }^{2}\right],$$

where the second equality follows from the energy-preserving property of the Fourier transform, similar to Eq. (40).

### KLD formulation for sensing-security metric

To characterize detection performance, we adopt the KLD as the metric. Our focus is on secure sensing in ISAC systems, where we aim to maximize the detection KLD at the BS to ensure reliable operation while simultaneously minimizing the detection KLD at Eve to suppress unauthorized sensing.

According to the system model, there are *K* targets in the scenario. Therefore, the hypothesis test for target detection includes the noise component and sidelobe interference generated by other targets. As justified in Supplementary Note 1, the aggregate sidelobe interference can be approximated as zero-mean CSCG due to the summation of independently sampled sidelobe contributions with different phase rotations from multiple interfering targets, with its variance characterized by the ISL-based interference power. Consequently, the effective noise variance comprises both the ISL-based sidelobe-interference power and the receiver’s noise. Under this effective noise model, the binary hypothesis test for target detection in a given DD bin (*l*, *k*) is formulated as follows:

Taking the detection hypothesis of target-*p* as an example, we henceforth omit the target index *p* without loss of generality. For the BS,

53 $${\left[{\widehat{{\mathbf{\Lambda }}}}_{{\rm{BS}}}\right]}_{l,k}=\left\{\begin{array}{ll}{\left[{\widehat{{\mathbf{\Lambda }}}}_{{\bf{I}}}^{{\rm{BS}}}\right]}_{l,k}+{\left[{\widehat{{\mathbf{\Lambda }}}}_{{\bf{Z}}}^{{\rm{BS}}}\right]}_{l,k},\quad &{{\mathcal{H}}}_{0,\mathrm{BS}},\\ {\left[{\widehat{{\mathbf{\Lambda }}}}_{{\bf{S}}}^{{\rm{BS}}}\right]}_{l,k}+{\left[{\widehat{{\mathbf{\Lambda }}}}_{{\bf{I}}}^{{\rm{BS}}}\right]}_{l,k}+{\left[{\widehat{{\mathbf{\Lambda }}}}_{{\bf{Z}}}^{{\rm{BS}}}\right]}_{l,k},\quad &{{\mathcal{H}}}_{1,\mathrm{BS}},\end{array}\right.$$

where *l* = 0,⋯, *M* − 1 and *k* = 0,⋯, *N* − 1. And for Eve,

54 $${\left[{\widehat{{\mathbf{\Lambda }}}}_{{\rm{E}}}\right]}_{l,k}=\left\{\begin{array}{ll}{\left[{\widehat{{\mathbf{\Lambda }}}}_{{\bf{I}}}^{{\rm{E}}}\right]}_{l,k}+{\left[{\widehat{{\mathbf{\Lambda }}}}_{{\bf{Z}}}^{{\rm{E}}}\right]}_{l,k},\quad &{{\mathcal{H}}}_{0,\mathrm{E}},\\ {\left[{\widehat{{\mathbf{\Lambda }}}}_{{\bf{S}}}^{{\rm{E}}}\right]}_{l,k}+{\left[{\widehat{{\mathbf{\Lambda }}}}_{{\bf{I}}}^{{\rm{E}}}\right]}_{l,k}+{\left[{\widehat{{\mathbf{\Lambda }}}}_{{\bf{Z}}}^{{\rm{E}}}\right]}_{l,k},\quad &{{\mathcal{H}}}_{1,\mathrm{E}},\end{array}\right.$$

where *l* = 0,⋯, *M* − 1 and *k* = 0,⋯, *N* − 1. Therefore, using the norm-squared filtered signal with the generalized likelihood ratio test (GLRT) detector, where the cell-averaging constant false alarm rate (CA-CFAR) detector used in the simulations can be regarded as a general extension of the GLRT when the effective noise power is unknown to the detector, the received signal in the RD map is defined as ${T}_{{\rm{BS}}}\triangleq {\left\vert {\left[{\widehat{{\mathbf{\Lambda }}}}_{{\rm{BS}}}\right]}_{l,k}\right\vert }^{2}$ and ${T}_{{\rm{E}}}\triangleq {\left\vert {\left[{\widehat{{\mathbf{\Lambda }}}}_{{\rm{E}}}\right]}_{l,k}\right\vert }^{2}$.

**Fig. 2:**
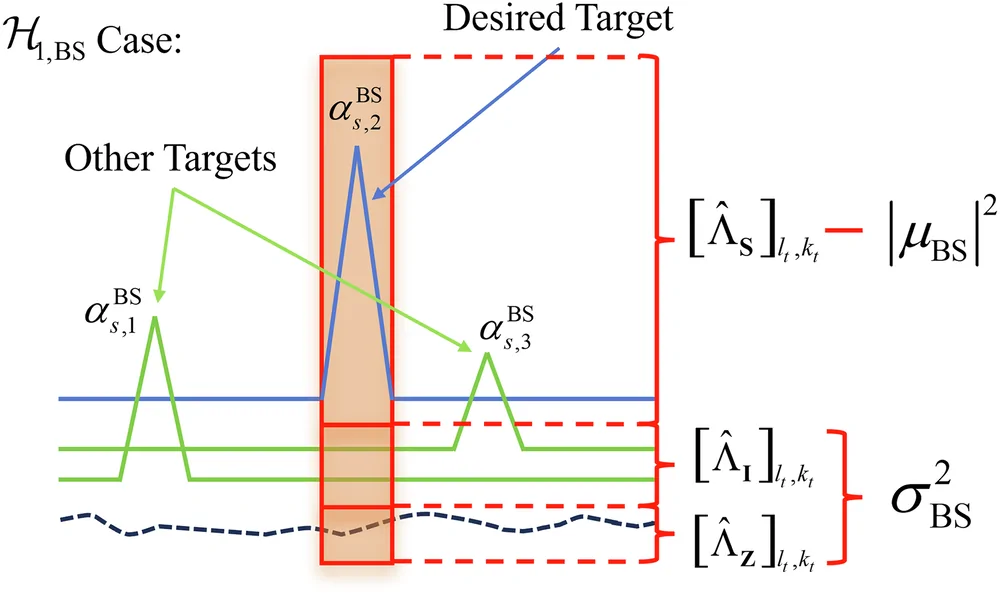
Kullback-Leibler divergence components for local target detection. The figure illustrates the components under ${{\mathcal{H}}}_{1,{\rm{BS}}}$ at the BS using a simplified one-dimensional sensing axis, which can be interpreted as either the delay or Doppler dimension. The filtered range-Doppler (RD)-domain response includes the desired target shown in blue, other-target interference shown in green, and the noise level indicated by the black dashed line, with analogous behavior in the two-dimensional delay-Doppler domain.

Given the CSCG model for the aggregate sidelobe interference, the probability distribution of the received signal at the BS’s and Eve’s sides can be expressed as follows. In the ${{\mathcal{H}}}_{0}$ case:

55 $${T}_{{\rm{BS}}} \sim \mathrm{Exp}\,\left({\sigma }_{\,\mathrm{BS}}^{2}\right),{T}_{{\rm{E}}} \sim \mathrm{Exp}\,\left({\sigma }_{\,\mathrm{E}\,}^{2}\right),$$

where ${\sigma }_{\,\mathrm{BS}}^{2}\triangleq {P}_{i,p,\mathrm{BS}}+{P}_{n,\mathrm{BS}}$, and ${\sigma }_{\,\mathrm{E}}^{2}\triangleq {P}_{i,p,\mathrm{E}}+{P}_{n,\mathrm{E}}$ are the effective noise variances for BS and Eve, respectively. Their PDFs are

56 $${f}_{0}\left({T}_{{\rm{BS}}}\right)=\frac{1}{{\sigma }_{\mathrm{BS}\,}^{2}}\exp \left(-\frac{{T}_{{\rm{BS}}}}{{\sigma }_{\mathrm{BS}\,}^{2}}\right),$$

57 $${f}_{0}\left({T}_{{\rm{E}}}\right)=\frac{1}{{\sigma }_{\mathrm{E}\,}^{2}}\exp \left(-\frac{{T}_{{\rm{E}}}}{{\sigma }_{\mathrm{E}\,}^{2}}\right),$$

where *T*_BS_ ≥ 0 and *T*_E_ ≥ 0. In the ${{\mathcal{H}}}_{1}$ case:

58 $$\frac{2}{{\sigma }_{\,\mathrm{BS}\,}^{2}}\,{T}_{{\rm{BS}}} \sim {\chi }_{2}^{{\prime} 2}\,\left(\frac{2| {\mu }_{{\rm{BS}}}{| }^{2}}{{\sigma }_{{\rm{BS}}}^{2}}\right),\frac{2}{{\sigma }_{\mathrm{E}\,}^{2}}\,{T}_{{\rm{E}}} \sim {\chi }_{2}^{{\prime} 2}\,\left(\frac{2| {\mu }_{{\rm{E}}}{| }^{2}}{{\sigma }_{\mathrm{E}\,}^{2}}\right),$$

where ${\chi }_{2}^{{\prime} 2}(\cdot )$ denotes the non-central chi-square distribution with two degrees of freedom, *μ*_BS_ = *μ*_*p*,BS_ and *μ*_E_ = *μ*_*p*,E_. Figure 2 illustrates the KLD components at the BS under ${{\mathcal{H}}}_{1,\mathrm{BS}}$: the effective noise (${\sigma }_{\,\mathrm{BS}\,}^{2}$) comprises noise and interference, and the mean (*μ*_BS_) corresponds to the desired target’s mainlobe. An analogous description applies to Eve under ${{\mathcal{H}}}_{1,\mathrm{E}}$. Accordingly, their probability distributions are

59 $${f}_{1}\left({T}_{{\rm{BS}}}\right)=\frac{1}{{\sigma }_{\mathrm{BS}\,}^{2}}\exp \left(-\frac{{T}_{{\rm{BS}}}+{\left\vert {\mu }_{{\rm{BS}}}\right\vert }^{2}}{{\sigma }_{\mathrm{BS}\,}^{2}}\right){I}_{0}\left(\frac{2\left\vert {\mu }_{{\rm{BS}}}\right\vert \sqrt{{T}_{{\rm{BS}}}}}{{\sigma }_{\mathrm{BS}\,}^{2}}\right),$$

60 $${f}_{1}\left({T}_{{\rm{E}}}\right)=\frac{1}{{\sigma }_{\mathrm{E}\,}^{2}}\exp \left(-\frac{{T}_{{\rm{E}}}+{\left\vert {\mu }_{{\rm{E}}}\right\vert }^{2}}{{\sigma }_{\mathrm{E}\,}^{2}}\right){I}_{0}\left(\frac{2\left\vert {\mu }_{{\rm{E}}}\right\vert \sqrt{{T}_{{\rm{E}}}}}{{\sigma }_{\mathrm{E}\,}^{2}}\right),$$

where *T*_BS_ ≥ 0, *T*_E_ ≥ 0, and both follow non-central chi-square distributions, and *I*_0_(⋅) denotes the zeroth-order modified Bessel functions of the first kind. Therefore, the KLD for the BS can be written as

61 $${D}_{{\rm{BS}}}=\int_{0}^{\infty }{f}_{1}(T)\,\ln \frac{{f}_{1}(T)}{{f}_{0}(T)}\,\,{\mathrm{d}}\,T$$

62 $$\begin{array}{ll}&=\int_{0}^{\infty }\frac{1}{{\sigma }_{\,\mathrm{BS}\,}^{2}}\exp \left(-\frac{T+| {\mu }_{{\rm{BS}}}{| }^{2}}{{\sigma }_{\mathrm{BS}\,}^{2}}\right){I}_{0}\,\left(\frac{2| {\mu }_{{\rm{BS}}}| \sqrt{T}}{{\sigma }_{\mathrm{BS}\,}^{2}}\right)\\ &\times \ln \left[\exp \left(-\frac{| {\mu }_{{\rm{BS}}}{| }^{2}}{{\sigma }_{\mathrm{BS}\,}^{2}}\right){I}_{0}\,\left(\frac{2| {\mu }_{{\rm{BS}}}| \sqrt{T}}{{\sigma }_{\mathrm{BS}\,}^{2}}\right)\right]\,\,{\mathrm{d}}\,T\end{array}$$

63 $$={{\mathbb{E}}}_{T \sim {f}_{1}}\,\left[\ln {I}_{0}\,\left(\frac{2| {\mu }_{{\rm{BS}}}| \sqrt{T}}{{\sigma }_{{\rm{BS}}}^{2}}\right)\right]-\frac{| {\mu }_{{\rm{BS}}}{| }^{2}}{{\sigma }_{\mathrm{BS}\,}^{2}}.$$

The KLD for Eve has a similar expression as

64 $${D}_{{\rm{E}}}({{\mathcal{H}}}_{1}\parallel {{\mathcal{H}}}_{0})={{\mathbb{E}}}_{T \sim {f}_{1}}\,\left[\ln {I}_{0}\,\left(\frac{2| {\mu }_{{\rm{E}}}| \sqrt{T}}{{\sigma }_{{\rm{E}}}^{2}}\right)\right]-\frac{| {\mu }_{{\rm{E}}}{| }^{2}}{{\sigma }_{\mathrm{E}\,}^{2}}.$$

Note that, ${D}_{{\rm{BS}}}({{\mathcal{H}}}_{1}\parallel {{\mathcal{H}}}_{0})$ and ${D}_{{\rm{E}}}({{\mathcal{H}}}_{1}\parallel {{\mathcal{H}}}_{0})$ are functions of ${\left[{w}_{1},{w}_{2},\cdots ,{w}_{MN}\right]}^{T}$, given a specific data sequence. Consequently, secure sensing can be achieved by maximizing the KLD at the BS while minimizing it at Eve.

The proposed KLD-based detection analysis is formulated for a given RD bin and serves as a local per-bin separability measure. It characterizes the statistical distinguishability between the target-present and target-absent hypotheses at the considered bin, capturing the effects of the desired mainlobe component, sidelobe interference, and noise on local target detectability. This formulation is consistent with the considered uniform target-search setting, where no specific DD-domain target-location prior is assumed. Under this setting, all candidate RD bins are treated equivalently, and the per-bin KLD provides a tractable surrogate for the global RD-map sensing-security behavior. With target-location prior information, the framework can be extended to a global or prior-weighted KLD over the full RD map to control competing peaks, false alarms, and localization ambiguity more directly.

Additionally, the derived KLD can be approximated for better tractability as

65 $$\begin{array}{ll}{\widetilde{D}}_{{\rm{BS}}}&\triangleq -\frac{| {\mu }_{{\rm{BS}}}{| }^{2}}{{\sigma }_{\mathrm{BS}\,}^{2}}+\ln \left\{{I}_{0}\left[\frac{2| {\mu }_{{\rm{BS}}}| }{{\sigma }_{\mathrm{BS}\,}^{2}}\,\sqrt{{\sigma }_{\,\mathrm{BS}\,}^{2}}\,\frac{\sqrt{\pi }}{2}\,\exp \left(-\frac{| {\mu }_{{\rm{BS}}}{| }^{2}}{2{\sigma }_{\mathrm{BS}\,}^{2}}\right)\right.\right.\\ &\left.\left.\times \left(\left(1+\frac{| {\mu }_{{\rm{BS}}}{| }^{2}}{{\sigma }_{\mathrm{BS}\,}^{2}}\right){I}_{0}\,\left(\frac{| {\mu }_{{\rm{BS}}}{| }^{2}}{2{\sigma }_{{\rm{BS}}}^{2}}\right)+\frac{| {\mu }_{{\rm{BS}}}{| }^{2}}{{\sigma }_{\mathrm{BS}\,}^{2}}{I}_{1}\,\left(\frac{| {\mu }_{{\rm{BS}}}{| }^{2}}{2{\sigma }_{\mathrm{BS}\,}^{2}}\right)\right)\right]\right\},\end{array}$$

which is detailed in Supplementary Note 2. Here, *I*_1_(⋅) denotes the first-order modified Bessel functions of the first kind. For the eavesdropper case, the approximated KLD is obtained by rewriting the expressions in terms of Eve’s parameters. Finally, we define the KLD gap as $\frac{{\widetilde{D}}_{{\rm{BS}}}}{{\widetilde{D}}_{{\rm{E}}}}$ for the sensing-security metric.

In this section, we formulate the optimization problem for the sensing-secure ISAC signaling design, followed by the optimization method that solves the proposed problem using the SA algorithm.

### Optimization problem

As discussed previously, securing the sensing functionality requires maximizing BS’s KLD and minimizing Eve’s KLD, thereby making the hypothesis test more distinguishable at the BS and less distinguishable at Eve. Consequently, the optimization problem for ISAC sensing-security can be formulated as follows:

66 $$\,\mathrm{max}\,{{\bf{W}}}_{{\rm{TF}}}\quad \beta \left[\frac{{\widetilde{D}}_{{\rm{BS}}}\left({{\bf{W}}}_{{\rm{TF}}}\right)}{{\widetilde{D}}_{{\rm{E}}}\left({{\bf{W}}}_{{\rm{TF}}}\right)}\right]-(1-\beta )\,{\left\Vert {{\bf{H}}}_{\mathrm{c},\mathrm{eff}}{{\bf{W}}}_{{\rm{TF}}}{\bf{U}}{\bf{s}}-{\bf{s}}\right\Vert }_{2}^{2}$$

67 $$\,\mathrm{s.t.}\,\,\,\,\,{\rm{Tr}}\left({{\bf{W}}}_{\,\mathrm{TF}\,}^{H}{{\bf{W}}}_{{\rm{TF}}}\right)\le {P}_{\max },$$

where *β* is the trade-off weighting factor, and **W**_TF_ is the TF perturbation matrix, providing joint amplitude and phase control as mentioned previously. Note that this optimization is formulated for a specific data sequence, i.e., a data-sequence-level optimization, and assumes the communication channel matrix is known at the BS.

We note that the above optimization allows for both Eve-aware and Eve-agnostic designs. Indeed, ${\widetilde{D}}_{{\rm{E}}}$ is defined in terms of Eve’s channel coefficients and noise power. If these quantities are known to the BS, this allows an Eve-aware approach. In practical implementations, however, the BS does not always have Eve’s exact parameters: these quantities can be assigned nominal Eve parameters, and the proposed method therefore remains applicable in Eve-agnostic scenarios. Evidently, in this case, the achievable security performance may result in a different sensing-security–communication trade-off than the ideal Eve-aware case, while any additional information about Eve, if available, can be incorporated into these parameters.

Moreover, ${\widetilde{D}}_{{\rm{E}}}$ is evaluated under the ideal assumption of a perfect reference signal at Eve, and thus represents an optimistic case on Eve’s sensing performance and a conservative (worst-case) scenario from the BS perspective.

### Optimization solution

The problem Eq. (66) is non-convex and exhibits a complex structure. Although the approximated KLDs, ${\widetilde{D}}_{{\rm{BS}}}$ and ${\widetilde{D}}_{{\rm{E}}}$, are convex with respect to (w.r.t.) $\frac{| {\mu }_{{\rm{BS}}}{| }^{2}}{{\sigma }_{\mathrm{BS}\,}^{2}}$ and $\frac{| {\mu }_{{\rm{E}}}{| }^{2}}{{\sigma }_{\mathrm{E}\,}^{2}}$, respectively, the intricate functional dependencies of *μ*_BS_, *μ*_E_, ${\sigma }_{\,\mathrm{BS}\,}^{2}$, and ${\sigma }_{\,\mathrm{E}\,}^{2}$ on ${\left[{w}_{1},\cdots ,{w}_{MN}\right]}^{T}$ further complicate the structure of problem Eq. (66). Therefore, we adopt the SA algorithm to solve the problem Wq. (66).

The SA algorithm is a well-established optimization method for tackling nonconvex problems and obtaining high-quality approximate solutions^47,48^. Starting from an initial solution, SA iteratively generates candidate solutions and, under a gradually decreasing temperature parameter, accepts improving moves while occasionally accepting deteriorating ones, thereby escaping local optima and enhancing global exploration. As the temperature is lowered, the search gradually shifts from global exploration to local exploitation, and the algorithm typically converges to a near-optimal solution^48^. Convex optimization may be a promising area for future research in solving this problem.

## Results

In this section, we present numerical results to validate the proposed sensing-security scheme for ISAC. Firstly, under the Eve-aware scenario, the detection probability for BS and Eve is evaluated, and illustrations comparing the DD responses with and without the sensing-security design are provided. In addition, a sensing case is investigated to further demonstrate the security effect by showing the different RD maps of BS and Eve. The trade-off between sensing-security and communication is then evaluated under different levels of multi-target sidelobe interference. This shows how the optimization allocates resources between the two functionalities in Eve-aware scenarios. Lastly, we present the comparison of the proposed method for Eve-aware and Eve-agnostic cases.

OTFS or OFDM with a given data sequence is assumed to be transmitted by the ISAC BS, while the sensing eavesdropper first extracts the reference signal from their direct link, equalizes it to estimate the transmitted signal, and then performs a passive sensing process. Unless stated otherwise, the communication information is modulated with the 16-ary quadrature amplitude modulation (16QAM) constellation. The number of Monte Carlo realizations for symbol sequences and noise is set to 100. Detection is implemented using CA-CFAR with a constant threshold of 10 dB. Other experimental setups are summarized in Tables 1 and 3 for the first two experiments, respectively.

**Table 1:** Simulation parameters for sensing-security centric design

Parameter	Value
ISAC balance weight *β*	1
Number of subcarriers *M*	8
Number of time-slots *N*	8
Communication channel delay *l*_c_	2
Communication channel Doppler *k*_c_	3
Communication channel coefficient *α*_c_	1
Communication SNR *SNR*_c_	25 dB
Communication Rician factor *κ*_c_	10 dB
Number of targets *K*	3
Unintended-target coeff. range ${\alpha }_{\,\mathrm{s},q}^{\mathrm{BS}\,}$, ${\alpha }_{\,\mathrm{s},q}^{\mathrm{E}\,}$	−15 to 10 dB
Direct link SNR for Eve *SNR*_ref_	0 dB
Direct link Rician factor for Eve *κ*_ref_	0 dB

### Secure-sensing performance evaluation: eve-aware case

In this subsection, we present experimental results for the detection probability and KLD of BS and Eve, with and without the security design, to further demonstrate the sensing-security performance and its relationship with KLD. In addition, to highlight the key manipulations in the sensing-security design, we present the DD responses of the transmitted signal. Both OTFS and OFDM waveforms are applied to evaluate the performance. Channel coefficients of unintended targets decrease uniformly across the specified range (as shown in Table 1).

In the simulations, *α*_c_ = 1 denotes a normalized effective communication-channel gain, rather than a physical path-loss value tied to a specific link distance or transmit power. Under this normalization, transmit power, propagation loss, and noise power are reflected through the adopted SNR setting, while in practical systems *α*_c_ can be specified from the corresponding path-loss model and link budget. Moreover, the small number of subcarriers is used as a proof-of-concept setting to clearly demonstrate the AF shaping mechanism and keep the optimization tractable; nevertheless, the proposed formulation is not restricted to this scale and can be extended to larger OFDM/OTFS dimensions.

**Fig. 3:**
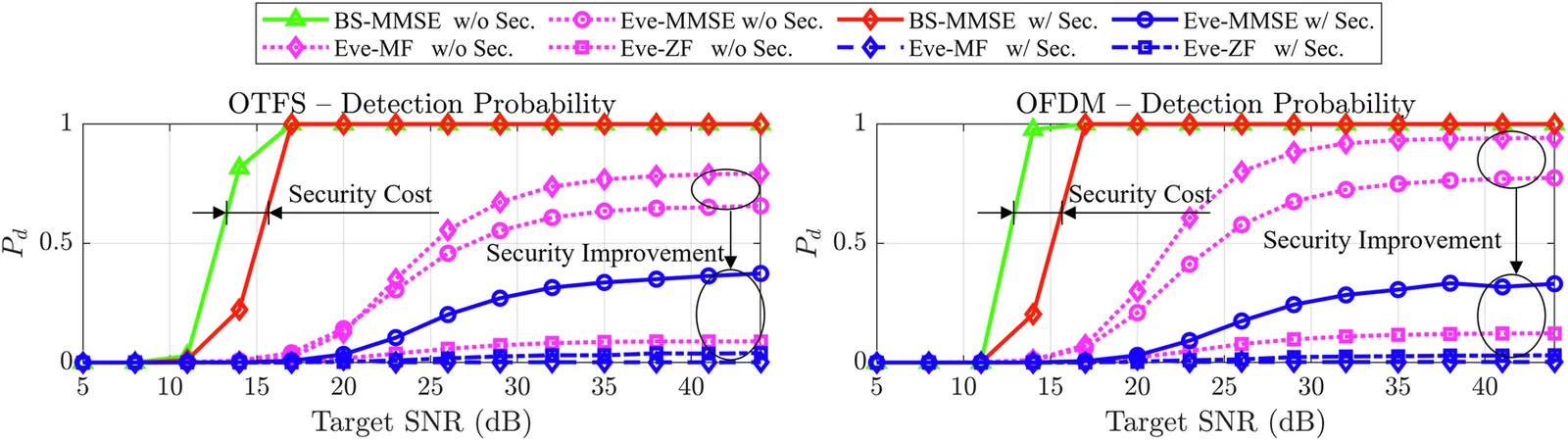
Detection probability curves of the BS and Eve under different sensing schemes. The figure reports the sensing-security-centric design with *β* = 1 and shows the detection probability curves of the BS and Eve for different sensing schemes.

**Fig. 4:**
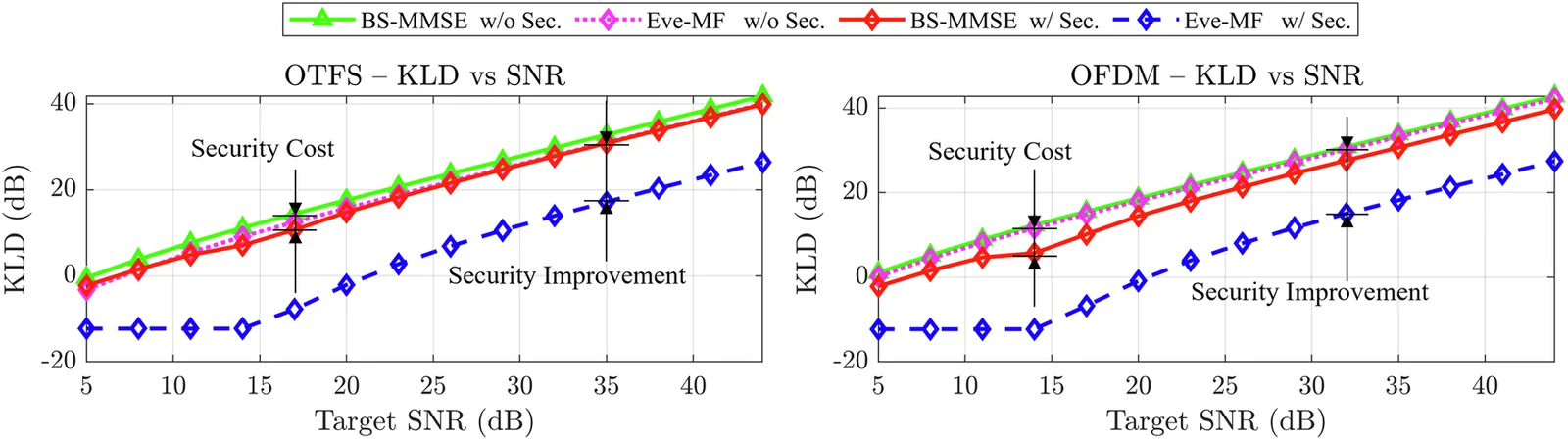
Kullback-Leibler divergence curves of the BS and Eve under different sensing schemes. The figure reports the sensing-security-centric design with *β* = 1 and shows the corresponding Kullback-Leibler divergence (KLD) curves.

The detection probability curves are illustrated in Fig. 3. It can be seen that the optimization algorithm effectively obtains the optimized solution of the sensing-security problem Eq. (66). The optimized results of the detection probability curves for both OTFS and OFDM waveforms demonstrate that the proposed sensing-security design effectively suppresses Eve’s detection probability, albeit with a slight offset in the detection probability of the BS. It is worth noting that, before the security design, the MF is the best filter for Eve, and it is the one most strongly suppressed by the proposed sensing-security processing. In addition, not only the MF, but also other filters, e.g., zero-forcing (ZF) and LMMSE, are suppressed by this method. Meanwhile, the optimized KLD curves in Fig. 4 also show that the proposed design effectively suppresses the KLD of Eve, albeit with a slight decrease in that of the BS.

**Fig. 5:**
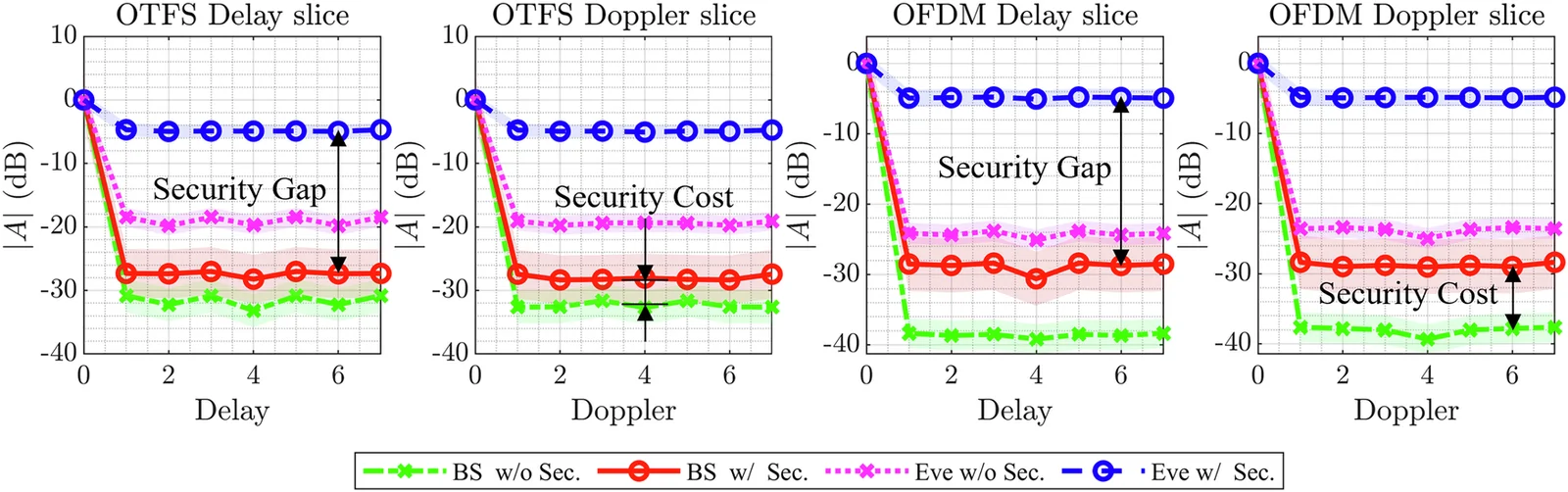
Averaged DD responses at the BS and Eve for a target at the zero-delay, zero-Doppler bin and an SNR of 10 dB. The figure shows delay and Doppler slices for both OTFS and OFDM waveforms with and without the proposed sensing-security optimization. The shaded regions qualitatively reflect the noise power for the corresponding methods, and the vertical separation between the BS and Eve curves reflects the DD response-based sensing-security margin.

Figure 5 illustrates the DD responses at the BS and Eve for a target at the zero-delay, zero-Doppler bin, evaluated at a representative target SNR of 10 dB. Delay and Doppler slices are shown for both OTFS and OFDM, each with and without the proposed sensing-security optimization. The optimal solution consistently raises Eve’s sidelobe floor for both waveforms, while the BS sidelobes remain clustered at much lower levels, with only a modest increase compared to the non-secure case. This vertical separation between the BS and Eve curves, highlighted as the “security gap” in the figure, can be interpreted as a DD response-based security margin: the BS retains a low-sidelobe DD response conducive to reliable detection, whereas Eve observes a significantly more distorted DD response with elevated sidelobes. Consequently, although the waveform is designed via a KLD-based criterion, its practical effect is to raise Eve’s effective interference level (or equivalently, the effective noise variance in the KLD), thereby degrading Eve’s detection probability while incurring only a limited sensing-performance cost at the BS. In practice, Eve may observe a target set different from that of the BS due to distinct sensing geometries and propagation conditions. The same-target-set setting in the Methods section is adopted to enable a controlled comparison and isolate the effect of the proposed waveform perturbation. Since the perturbation is imposed on the transmitted signal itself, Eve’s passive sensing performance is still expected to be impaired for different observed targets, as long as Eve relies on the BS-emitted signal.

Since the proposed perturbation is imposed in the TF domain, OFDM and OTFS exhibit generally similar optimized performance, as reflected by the KLD and detection-probability results in Figs. 3 and 4: OFDM is directly modulated in the TF domain, and OTFS is modulated in the DD domain and then transformed into the TF domain. Nevertheless, their different modulation-domain mappings lead to observable differences before optimization. Specifically, ISFFT in OTFS spreads DD-domain QAM symbols into the TF domain, causing stronger TF-domain power fluctuation than OFDM. According to Eqs (40) and (52), stronger TF-domain power fluctuation leads to a larger ISL and stronger sidelobe interference, as observed from the DD response results in Fig. 5. Therefore, OFDM exhibits lower sidelobe levels at both the BS and Eve than OTFS before optimization, leading to stronger Eve sensing capability and higher Eve-side KLD and detection probability, as shown in Figs. 3 and 4. After optimization, the TF-domain perturbation dominates the original modulation-induced fluctuation, making the OFDM and OTFS performance difference much less pronounced.

**Fig. 6:**
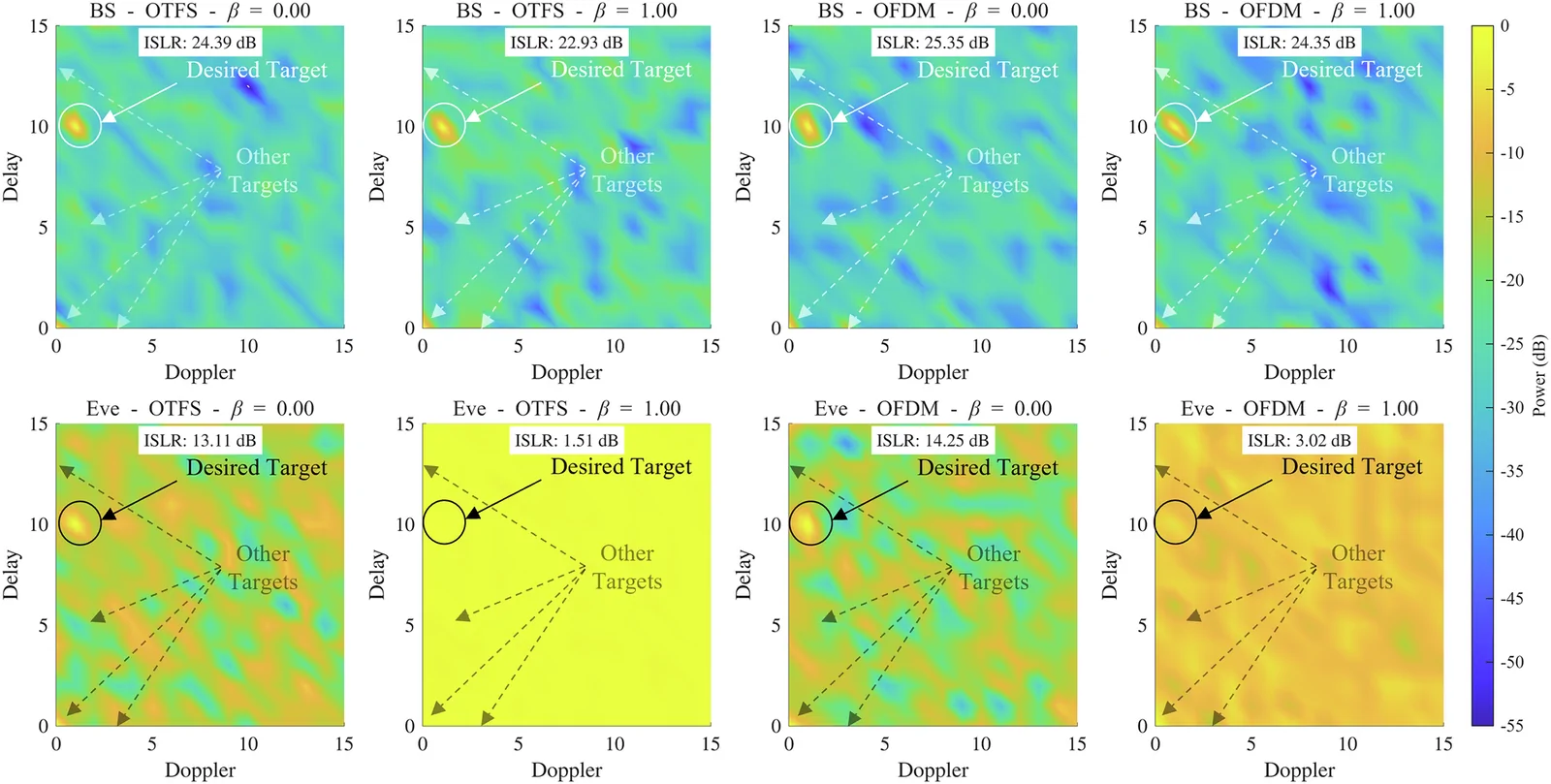
RD maps versus sensing-security preference for the BS and Eve. The figure shows optimized sensing-secured ISAC signals at a target SNR of 20 dB, with the BS results shown in the top panels and the Eve results shown in the bottom panels. The numbers of subcarriers and time slots are *M* = *N* = 16, and the number of targets is *K* = 5.

Figure 6 illustrates the RD maps of a specific case for the BS and Eve under different weighting preference factors, *β*, using a specific data sequence for transmission. For the communication-centric design (*β* = 0), the desired target exhibits high RD contrast at both nodes: the integrated sidelobe level ratio (ISLR) for the desired target at the BS is 24.39 dB and 25.35 dB for OTFS and OFDM, respectively, while Eve attains ISLR values of 13.11 dB for OTFS and 14.25 dB for OFDM, indicating that the target remains relatively well resolved. In contrast, for the sensing-security-centric design (*β* = 1), the BS still preserves a pronounced mainlobe with ISLR values of 22.93 dB for OTFS and 24.35 dB for OFDM, whereas Eve’s ISLR is drastically reduced to 1.51 and 3.02 dB, respectively, rendering the desired target barely distinguishable from the surrounding multi-target interference. These quantitative results substantiate that the proposed waveform design impairs Eve’s sensing capability while maintaining reliable target detection at the BS.

This behavior further indicates that the proposed design can remain effective for fractional DD targets, since the integer-grid DD responses are sampled points of the underlying continuous AF. Therefore, shaping the integer-grid sidelobe structure generally also affects neighboring continuous DD regions, making Eve’s off-grid target responses more likely to be contaminated by elevated sidelobes.

**Fig. 7:**
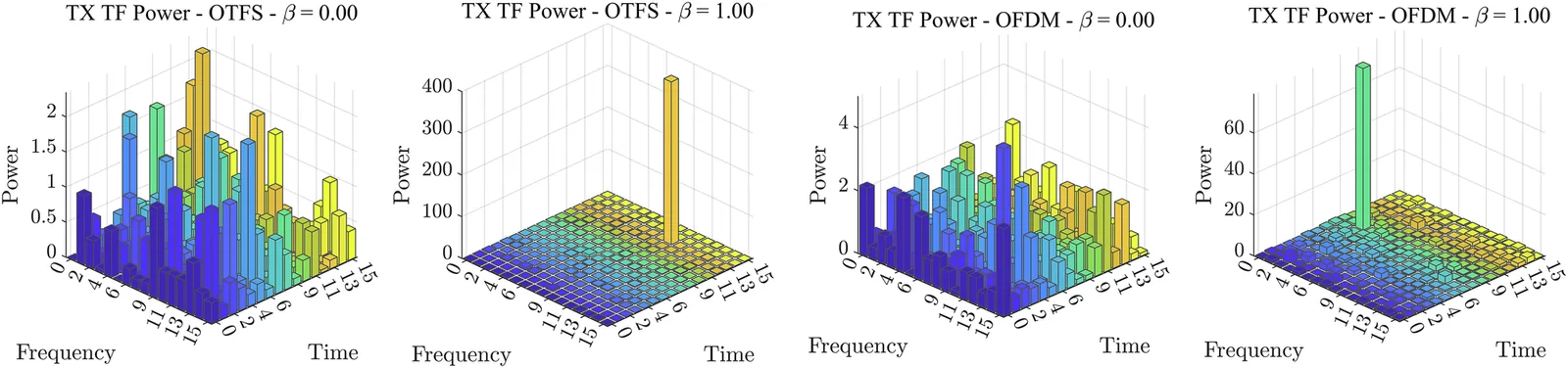
TF-domain power distribution of the transmitted signal versus security preference. The figure compares OTFS and OFDM waveforms at a target SNR of 20 dB, with *M* = *N* = 16 and *K* = 5. The results show how the optimized time-frequency (TF)-domain power distribution changes as the sensing-security preference varies.

Figure 7 illustrates the TF-domain power distributions of the transmitted OTFS and OFDM waveforms under *β* = 0 and *β* = 1. For *β* = 0, the transmit power is broadly spread over the TF grid, which is consistent with a communication-centric design. For *β* = 1, the optimization yields a highly concentrated TF power pattern, reflecting a clear trade-off between communication performance and sensing-security. Importantly, this concentration at the BS does not impair its sensing performance: under the considered high-SNR regime, the BS LMMSE processor operates close to a ZF receiver, effectively canceling the contributions of all transmit symbols. Hence, even though many TF samples exhibit low power, they still carry channel information that can be recovered after ZF, preserving the BS’s RD response structure. In contrast, Eve relies on an MF receiver, for which the concentrated transmit power leads to significant sidelobes in the RD map, thereby increasing interference and degrading Eve’s detection performance. In summary, communication favors spread randomness, sensing favors spread determinism^49^, whereas sensing-security favors concentrated determinism in the TF domain as shown in Fig. 7.

The results of Figs. 6 and 7 also support the effectiveness of the proposed KLD-based design, showing that the ISL-based power characterization can effectively guide the optimization to regulate Eve’s sensing performance.

**Fig. 8:**
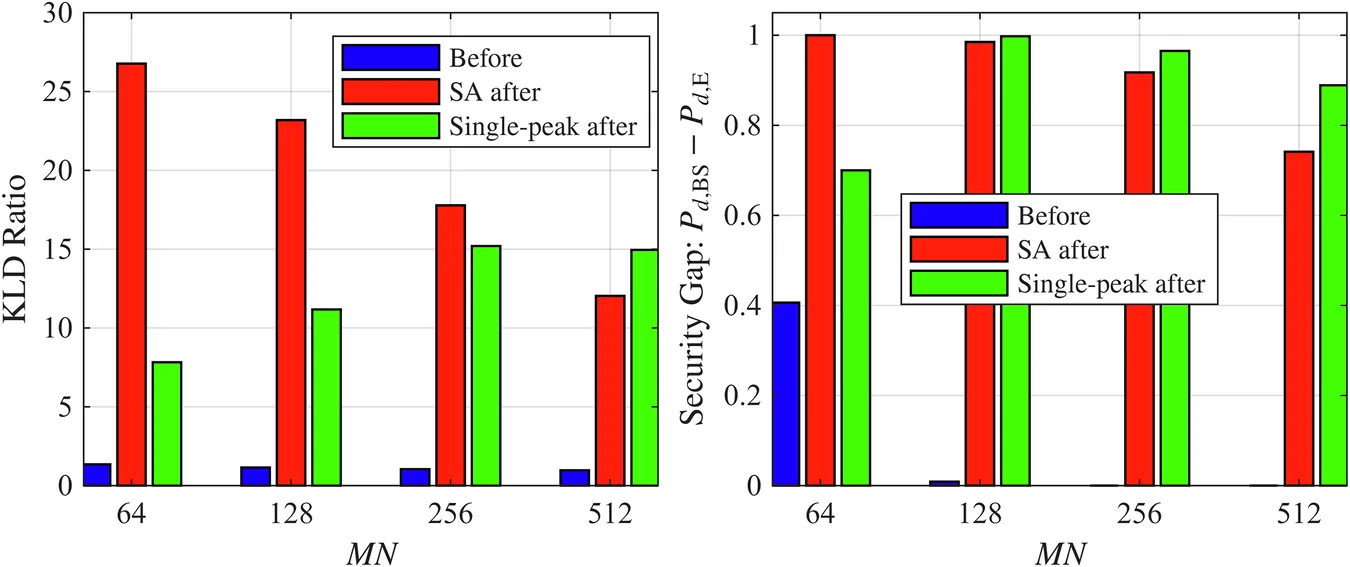
KLD ratio and detection-probability separation for the initial waveform, SA optimization, and single-peak principle. The figure compares the initial waveform, simulated annealing (SA) optimization, and the single-peak principle at a target SNR of 25 dB. The maximum number of SA iterations is 5 × 10^5^.

To further verify the necessity of the proposed optimization and its computational cost, we compare the SA-based optimization with a non-iterative single-peak principle, which directly assigns the dominant TF-domain power to the selected peak position while maintaining a small floor-power level over the remaining TF-domain bins. As shown in Table 2, the single-peak principle is much faster than SA because it avoids the iterative optimization procedure. However, Fig. 8 shows that SA achieves better performance in the low-dimensional cases, especially for smaller *MN*, since it directly optimizes the KLD-based objective. When *MN* becomes large, the SA performance degrades because the algorithm reaches the maximum number of iterations before sufficient convergence. Therefore, the single-peak principle provides a fast heuristic baseline, while the SA-based method remains more effective when the optimization problem size is moderate and sufficient convergence can be reached. Consequently, developing more efficient algorithms, such as efficient peak-location search, is a promising future direction.

**Table 2:** Average design time per symbol sequence of the SA optimization and the single-peak principle at a target SNR of 25 dB

*MN*	SA time (s)	Single-peak time (s)	Speedup
64	16.657	1.850 × 10^−3^	9.00 × 10^3^
128	43.889	5.817 × 10^−4^	7.55 × 10^4^
256	483.93	9.108 × 10^−4^	5.31 × 10^5^
512	4764.7	1.687 × 10^−3^	2.82 × 10^6^

### Sensing-secure ISAC trade-off: eve-aware case

In this subsection, we present the OTFS results to illustrate the trade-off between sensing-security and communication performance, since OFDM exhibits similar performance. The simulation parameters are summarized in Table 3. Specifically, we investigate the bottom-end metrics for sensing-security and communication-detection probability and average communication rate under various multi-target interference environments.

**Table 3:** Simulation parameters for the sensing-secured ISAC trade-off experiment

Parameter	Value
Number of subcarriers *M*	8
Number of time-slots *N*	8
Communication channel delay *l*_c_	2
Communication channel Doppler *k*_c_	3
Communication channel coefficient *α*_c_	1
Communication SNR *SNR*_c_	25 dB
Communication Rician factor *κ*_c_	10 dB
Number of targets *K*	3
Target SNR for BS and Eve *SNR*_s_	25 dB
Direct link SNR for Eve *SNR*_ref_	20 dB
Direct link Rician factor for Eve *κ*_ref_	10 dB

**Fig. 9:**
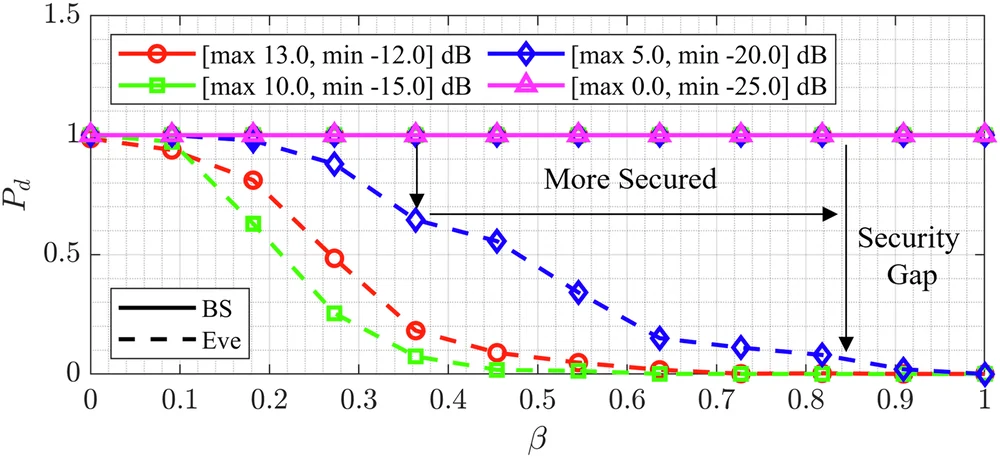
Detection probability trade-off transition in OTFS. The figure shows the detection probability trade-off transition under different scatter reflection conditions.

**Fig. 10:**
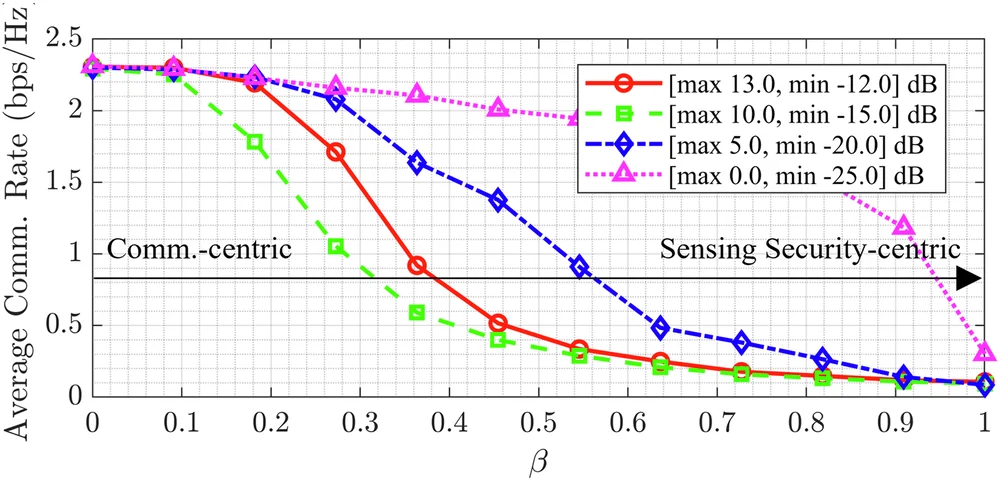
Average communication rate trade-off transition in OTFS. The figure shows the average communication rate trade-off transition under different scatter reflection conditions.

Figures 9 and 10 show the trade-off. The desired and other targets are considered in an environment with channel coefficients spanning a specified range and decreasing in uniform steps from the upper to the lower bound, as labeled in the figure. In Fig. 9, as the priority weight *β* increases from 0 to 1, the gap between the detection probabilities of BS and Eve widens: the detection probability of Eve decreases, while that of BS remains essentially unchanged. However, when the scatter-reflection power is sufficiently low, the security effect reflected in the detection probability vanishes, indicating that guaranteeing sensing security is challenging in near scatter-free environments. In addition, under the most severe multi-target interference conditions ([−12, 13] dB), security performance begins to improve later than in the [−15, 10] dB case, indicating that, with severe interference, the system can still achieve a secure design but finds it harder to enhance the BS sensing performance while degrading Eve’s sensing performance. As a consequence, sensing-security is difficult to realize under both extremely severe and relatively weak interference conditions, reflecting an inherent conflict between the BS and Eve: severe interference degrades Eve but also the BS, whereas weak interference improves the BS and likewise Eve; however, with proper exploitation of such interference, sensing-security can still be achieved. Figure 10 illustrates the communication performance under different priority weights and interference conditions, indicating that the security performance comes at the expense of communication degradation. In both low- and high-interference environments, the available degrees of freedom cannot be efficiently leveraged to enhance sensing-security—owing to the sensing-performance conflict between BS and Eve—so the optimization naturally prioritizes communication performance. Consequently, the pink curve encloses the largest communication-performance region. The red curve shifts left relative to the green, and the remaining curves are ordered to reflect progressively better communication performance as sensing interference decreases, indicating that the system tends to allocate more of the available degrees of freedom to communication performance.

**Fig. 11:**
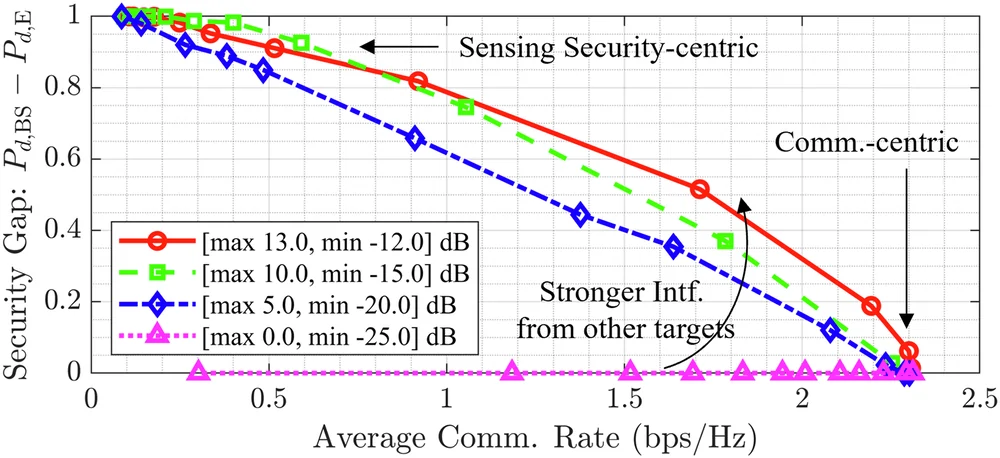
Trade-off between bottom-end sensing-security and communication metrics. The figure shows the detection probability difference between the BS and Eve and the average communication rate under different scatter reflection conditions for OTFS. The curves illustrate how the performance region changes with the level of multi-target interference.

Figure 11 illustrates the trade-off between bottom-end metrics, specifically the detection probability difference between the BS and Eve, and the average communication rate.

As multi-target interference increases, the performance region first expands and then saturates beyond a bottleneck, as shown by the green and red curves. Under extremely severe interference, the boundary further reshapes toward communication performance, since allocating excessive degrees of freedom to sensing-security brings limited additional gain.

The trade-off region provides a practical guideline for selecting *β*. When multi-target interference is weak, the reflected components are insufficient to create a clear sensing-security gap between the BS and Eve; hence, a smaller *β* is preferred to prioritize communication performance. For moderate interference, the trade-off is more balanced, and *β* can be chosen from a wider intermediate range according to the desired sensing-security–communication preference. As interference becomes stronger, a larger *β* is generally required to achieve the same sensing-security level, indicating that sensing-security design becomes more difficult because the interference suppresses Eve’s sensing capability while also degrading the BS sensing performance.

### Comparison between Eve-aware and Eve-agnostic cases

**Fig. 12:**
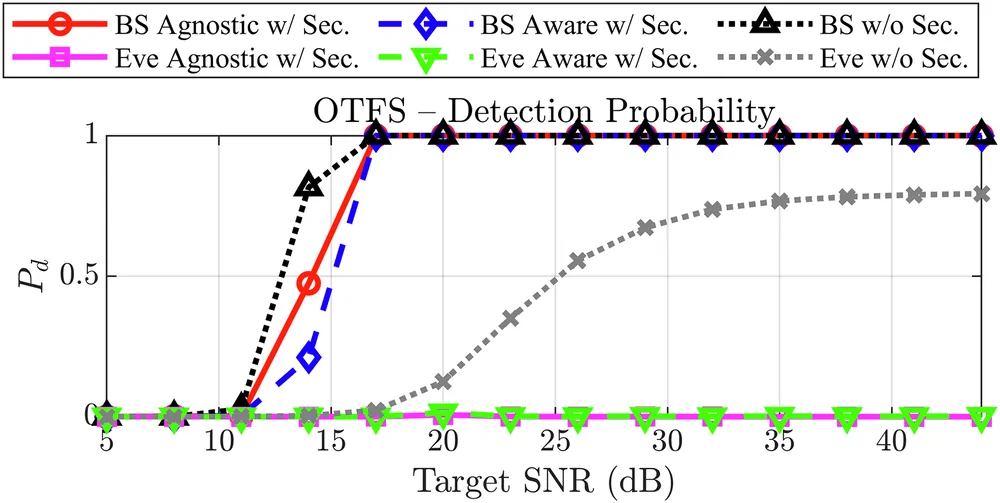
Comparison between Eve-aware and Eve-agnostic cases. The figure compares the performance of the proposed method for the OTFS waveform when Eve-related parameters are available and when nominal Eve parameters are used. The results illustrate the applicability of the proposed design in both Eve-aware and Eve-agnostic settings.

Figure 12 illustrates the applicability of the proposed method to both Eve-aware and Eve-agnostic scenarios, using the same experimental setup as in the secure-sensing performance evaluation. In the Eve-agnostic case, the BS does not rely on Eve’s exact channel, noise, or target-related parameters. Instead, nominal Eve parameters are used to construct the sensing-security metric. The effectiveness of this treatment depends mainly on whether the nominal parameters preserve representative effective power ratios among the desired mainlobe component, sidelobe/interference power, and noise power in Eve’s sensing observation. If these ratios are representative, the optimized waveform can still degrade Eve’s sensing quality by reshaping the AF sidelobe structure. The results show that the proposed method remains effective under this nominal-parameter setting, exhibiting only a slight performance difference at the BS compared with the Eve-aware design. Nevertheless, a rigorous theoretical characterization for general Eve realizations would require a robust formulation over an uncertainty set of Eve’s parameters, which is left as future work.

## Discussion

This paper investigated sensing-security for practical ISAC waveforms from a detection-oriented perspective. We established system models for UE communication, BS sensing, and Eve’s passive sensing, and analytically decomposed the RD-domain sensing response into mainlobe, sidelobe, and noise components for both BS and Eve. Building on this, we introduced KLD-based detection metrics and derived a tractable Jensen surrogate to facilitate the analysis and optimization. Leveraging this metric together with a communication-error penalty, we formulated a sensing-security optimization over TF-domain perturbations and solved the resulting nonconvex design via SA. Numerical results verified that the proposed method reliably suppresses Eve’s sensing by elevating their sidelobes and reducing their detection probability, while causing only a modest performance loss at the BS. The experiments further revealed a clear sensing-security-communication trade-off: stronger sensing-security lowers Eve’s capability at the cost of reduced rate and increased communication error. Overall, the proposed framework provided an effective and practical approach for enhancing sensing-security in ISAC systems.

Future work may first extend the proposed framework to joint pilot- and data-based unauthorized sensing. For pilot-based sensing, publicly known pilots may enable Eve’s sensing capability and limit the proposed protection. For data-based sensing, pilot-based equalization cannot fully remove the TF-domain perturbation, but the resulting residual sensing-security performance requires further characterization. In addition, full RD-map or prior-weighted KLD formulations are worth investigating to control false alarms, competing peaks, and localization ambiguity. Furthermore, robust Eve-agnostic design under channel, noise, target-parameter, and BS–Eve direct-link uncertainty is another important direction. Finally, practical extensions include more general target mobility models, explicit UE-side equalization/decoding, peak-to-average power ratio and power-amplifier constraints, and low-complexity optimization for large-scale ISAC systems.

## Supplementary information

Supplementary information

## Data Availability

The datasets generated and/or analyzed during the current study are not publicly available because this work is based on numerical simulations rather than a standalone curated dataset, but the numerical data underlying the figures are available from the corresponding author on reasonable request.

## Code availability

The code generated and analyzed during the current study is not publicly available due to its continued development for ongoing and follow-up research but is available from the corresponding author on reasonable request.

## References

[1] Liu, F., Masouros, C., Petropulu, A. P., Griffiths, H. & Hanzo, L. Joint radar and communication design: applications, state-of-the-art, and the road ahead. *IEEE Trans. Commun.* **68**, 3834–3862 (2020).

[2] Bazzi, A., Ying, M., Kanhere, O., Rappaport, T. S. & Chafii, M. ISAC imaging by channel state information using ray tracing for next generation 6G. *IEEE J. Sel. Top. Electromagn. Antennas Propag.* **1**, 135–149 (2025).

[3] Qu, K., Ye, J., Li, X. & Guo, S. Privacy and security in ubiquitous integrated sensing and communication: threats, challenges and future directions. *IEEE Internet Things Mag.* **7**, 52–58 (2024).

[4] Chen, J. et al. A survey on directional modulation: opportunities, challenges, recent advances, implementations, and future trends. *IEEE Internet Things J.* **12**, 32581–32615 (2025).

[5] Su, N. et al. Integrating sensing and communications in 6g? not until it is secure to do so. *IEEE Wireless Commun.* 10.1109/MWC.2026.3703015 (early access, 2026).

[6] Su, N., Liu, F. & Masouros, C. Sensing-assisted eavesdropper estimation: An ISAC breakthrough in physical layer security. *IEEE Trans. Wirel. Commun.* **23**, 3162–3174 (2024).

[7] Liu, Q. et al. Secure OTFS transmission via joint delay-doppler precoding and time-domain noise injection. In *Proc. IEEE/CIC ICCC Workshops*, 569–574 (IEEE, 2024).

[8] Zou, J., Masouros, C., Liu, F. & Sun, S. Securing the sensing functionality in ISAC networks: an artificial noise design. *IEEE Trans. Veh. Technol*. **73**, 17800–17805 (2024).

[9] Han, K., Meng, K. & Masouros, C. Sensing-secure ISAC: ambiguity function engineering for impairing unauthorized sensing. *IEEE Trans. Wireless Commun*. **25**, 5386–5400 (2025).

[10] Su, N., Liu, F. & Masouros, C. Secure radar-communication systems with malicious targets: Integrating radar, communications and jamming functionalities. *IEEE Trans. Wirel. Commun.* **20**, 83–95 (2021).

[11] Su, N., Liu, F., Wei, Z., Liu, Y.-F. & Masouros, C. Secure dual-functional radar-communication transmission: exploiting interference for resilience against target eavesdropping. *IEEE Trans. Wirel. Commun.* **21**, 7238–7252 (2022).

[12] Ghiro, L., Cominelli, M., Gringoli, F. & Lo Cigno, R. On the implementation of location obfuscation in openwifi and its performance. In *Proc. MedComNet*, 64–73 (IEEE, 2022).

[13] Abanto-Leon, L. F., Bäuml, A., Sim, G. H. A., Hollick, M. & Asadi, A. Stay connected, leave no trace: enhancing security and privacy in WiFi via obfuscating radiometric fingerprints. *Proc. ACM Meas. Anal. Comput. Syst.* **4**, 44:1–44:31 (2020).

[14] Qiao, Y., Zhang, O., Zhou, W., Srinivasan, K. & Arora, A. PhyCloak: obfuscating sensing from communication signals. In *Proc. USENIX NSDI*, 685–699 (USENIX Association, 2016).

[15] Jiang, J., Wang, J., Liu, Y., Chen, Y. & Liu, Y. WiCloak: protect location privacy of wifi devices. In *Proc. ACM/IEEE International Conference on Information Processing in Sensor Networks*, 101–112 (ACM, 2024).

[16] Meng, X. et al. Secur-Fi: a secure wireless sensing system based on commercial wi-fi devices. In *Proc. IEEE INFOCOM*, 1–10 (IEEE, 2023).

[17] Wang, Y., Sun, L. & Du, Q. Multi-antenna signal masking and round-trip transmission for privacy-preserving wireless sensing. *IEEE Trans. Inf. Forensics Security* **19**, 6305–6320 (2024).

[18] Hu, J. et al. WiShield: privacy against wi-fi human tracking. *IEEE J. Sel. Areas Commun.* **42**, 2970–2984 (2024).

[19] Luo, J., Cao, H., Jiang, H., Yang, Y. & Chen, Z. MIMOCrypt: multi-user privacy-preserving wi-fi sensing via MIMO encryption. In *Proc. IEEE Symposium on Security and Privacy*, 2812–2830 (IEEE, 2024).

[20] Staat, P. et al. IRShield: a countermeasure against adversarial physical-layer wireless sensing. In *Proc. IEEE**Symposium on Security and Privacy*, 1705–1721 (IEEE, 2022).

[21] Jiang, C. et al. RISiren: wireless sensing system attacks via metasurface. In *Proc. ACM SIGSAC Conference on Computer and Communications Security*, 3332–3345 (ACM, 2024).

[22] Chen, Y. & Wang, J. ScatterShield: defending against unauthorized wifi sensing with backscatter tags. *Proc. ACM Interact. Mob. Wearable Ubiquitous Technol.* **9**, 166:1–166:24 (2025).

[23] Guo, T. & Li, H. Secure integrated sensing and communications (s-ISAC) network. In *Proc. IEEE VTC-Fall*, 1–6 (IEEE, 2024).

[24] Han, K., Masouros, C., Riihonen, T. & Amin, M. G. Next-generation MIMO transceivers for integrated sensing and communications: unique security vulnerabilities and solutions. *Proc. IEEE* **114**, 18–51 (2026).

[25] Li, X. et al. Win-win of communication & sensing security for MC-NOMA ISAC systems. *IEEE J. Sel. Areas Commun*. **43**, 3214–3230 (2025).

[26] Wang, J. et al. Generative AI based secure wireless sensing for ISAC networks. *IEEE Trans. Inf. Forensics Secur.* **20**, 5195–5210 (2025).

[27] Chen, J. et al. Sensing security in near-field ISAC: Exploiting scatterers for eavesdropper deception. Preprint at 10.48550/arXiv.2510.20140 (2025).

[28] Bönsch, J., Reitz, P., Franchi, N. & Lübke, M. Evaluating communication and sensing quality in phase-modulated FMCW radar. In *Proc. IEEE Joint Communications & Sensing Symposium*, 1–6 (IEEE, 2025).

[29] Hadani, R. et al. Orthogonal time frequency space modulation. In *Proc. IEEE WCNC*, 1–6 (IEEE, 2017).

[30] Yuan, W., Wei, Z., Li, S., Yuan, J. & Ng, D. W. K. Integrated sensing and communication-assisted orthogonal time frequency space transmission for vehicular networks. *IEEE J. Sel. Top. Signal Process.* **15**, 1515–1528 (2021).

[31] Bemani, A., Ksairi, N. & Kountouris, M. AFDM: a full diversity next generation waveform for high mobility communications. In *Proc. IEEE ICC Workshops*, 1–6 (IEEE, 2021).

[32] Bemani, A., Ksairi, N. & Kountouris, M. Integrated sensing and communications with affine frequency division multiplexing. *IEEE Wirel. Commun. Lett.* **13**, 1255–1259 (2024).

[33] Liu, F. et al. Uncovering the iceberg in the sea: fundamentals of pulse shaping and modulation design for random ISAC signals. *IEEE Trans. Signal Process.* **73**, 2511–2526 (2025).

[34] Chong, R. et al. Delay-Doppler ISAC: ambiguity function analysis via zak-OTFS modulation. In *Proc. IEEE GLOBECOM* (IEEE, 2025).

[35] Bedeer, E. Ambiguity function analysis of affine frequency division multiplexing for integrated sensing and communication. In *IEEE J. Sel. Areas Commun.* **44**, 196–211 (2026).

[36] Raviteja, P., Phan, K. T., Hong, Y. & Viterbo, E. Orthogonal time frequency space (OTFS) modulation based radar system. In *Proc. IEEE Radar Conference*, 1–6 (IEEE, 2019).

[37] Palmer, J. E. & Searle, S. J. Evaluation of adaptive filter algorithms for clutter cancellation in passive bistatic radar. In *Proc. IEEE Radar Conference*, 493–498 (IEEE, 2012).

[38] Colone, F., O’Hagan, D. W., Lombardo, P. & Baker, C. J. A multistage processing algorithm for disturbance removal and target detection in passive bistatic radar. *IEEE Trans. Aerosp. Electron. Syst.* **45**, 698–722 (2009).

[39] Raviteja, P., Hong, Y., Viterbo, E. & Biglieri, E. Practical pulse-shaping waveforms for reduced-cyclic-prefix OTFS. *IEEE Trans. Veh. Technol.* **68**, 957–961 (2019).

[40] Zhang, K., Yuan, W., Fan, P. & Wang, X. Dual-functional waveform design with local sidelobe suppression via OTFS signaling. *IEEE Trans. Veh. Technol.* **73**, 14044–14049 (2024).

[41] Song, G. et al. Low sidelobe level and PAPR OTFS waveform design for ISAC systems. *IEEE Trans. Commun.* **73**, 7952–7966 (2025).

[42] Keskin, M. F. et al. Fundamental trade-offs in monostatic ISAC: a holistic investigation toward 6G. *IEEE Trans. Wirel. Commun.* **24**, 7856–7873 (2025).

[43] Lingadevaru, P., Pardhasaradhi, B., Srihari, P. & Sharma, G. V. K. Analysis of 5g new radio waveform as an illuminator of opportunity for passive bistatic radar. In *Proc. IEEE NCC*, 1–6 (IEEE, 2021).

[44] Ai, X., Zhang, L., Zheng, Y. & Zhao, F. Passive detection experiment of UAV based on 5g new radio signal. In *Proc. PIERS*, 2124–2129 (IEEE, 2021).

[45] Samczyński, P. et al. 5g network-based passive radar. *IEEE Trans. Geosci. Remote Sens.* **60**, 1–9 (2022).

[46] Abratkiewicz, K. et al. SSB-based signal processing for passive radar using a 5g network. *IEEE J. Sel. Top. Appl. Earth Obs. Remote Sens.* **16**, 3469–3484 (2023).

[47] Ingber, L. Simulated annealing: practice versus theory. *Math. Comput. Model.* **18**, 29–57 (1993).

[48] Delahaye, D., Chaimatanan, S. & Mongeau, M. Simulated annealing: From basics to applications. In (eds Gendreau, M. & Potvin, J.-Y.) *Handbook of Metaheuristics*, vol. 272 of *International Series in Operations Research & Management Science*, 1–35 (Springer, Cham, 2019).

[49] Liu, F., Xiong, Y., Wan, K., Han, T. X. & Caire, G. Deterministic–random tradeoff of integrated sensing and communications in Gaussian channels: a rate–distortion perspective. In *Proc. 2023 IEEE International Symposium on Information Theory (ISIT)*, 2326–2331 (IEEE, 2023).

