# Supplementary material for: Securing the sensing functionality in ISAC: KLD-based ambiguity function shaping
Source: NPJ Wirel Technol. 2026 Oct 1;2(1):79. doi: 10.1038/s44459-026-00089-0 (PMC13630560; doi:10.1038/s44459-026-00089-0)
Supplement: Supplementary file 1 [file 44459_2026_89_MOESM1_ESM.pdf]

Supplementary Information for Securing the Sensing Functionality in ISAC: KLD-Based Ambiguity Function Shaping

Borui Du, Kawon Han, and Christos Masouros

Supplementary Note 1. Justification of the CSCG Sidelobe-Interference Model

This Supplementary Note justifies the CSCG approximation of the sidelobe interference used in the Methods section of the main manuscript. We write the derivation for a generic receiver $\eta \in \{\mathrm{BS}, \mathrm{E}\}$. The BS-side LMMSE receiver and the Eve-side MF receiver are obtained by substituting their corresponding TF-domain weights.

For compactness, define the receiver-dependent TF-domain weighting as

$$\gamma_{m,n}^{\eta} = \begin{cases} \dfrac{|x_{m,n}|^2}{|x_{m,n}|^2 + \sigma^2_{\mathbf{z}_{\mathrm{BS,s}}}}, & \eta = \mathrm{BS}, \\ |x_{m,n}|^2, & \eta = \mathrm{E}. \end{cases} \quad \text{(S1)}$$

Thus, $\gamma_{m,n}^{\mathrm{BS}}$ corresponds to the BS LMMSE receiver in (40), while $\gamma_{m,n}^{\mathrm{E}}$ corresponds to the Eve MF receiver in (52).

A. Zero-Mean Property of the Off-Mainlobe Response

For a relative DD offset $(\Delta l, \Delta k)$, define

$$\chi_{m,n}(\Delta l, \Delta k) \triangleq \exp\left\{ j2\pi \left(\tfrac{m\Delta l}{M} - \tfrac{n\Delta k}{N} \right) \right\}. \quad \text{(S2)}$$

The receiver-dependent RD response is then written as

$$\left[\widetilde{\mathbf{\Lambda}}_{\eta}\right]_{\Delta l, \Delta k} = \frac{1}{\sqrt{MN}} \sum_{m=0}^{M-1} \sum_{n=0}^{N-1} \gamma_{m,n}^{\eta} \chi_{m,n}(\Delta l, \Delta k). \quad \text{(S3)}$$

The mainlobe corresponds to $(\Delta l, \Delta k) = (0,0)$, while $(\Delta l, \Delta k) \neq (0,0)$ gives an off-mainlobe response.

For a fixed transmitted block, $\gamma_{m,n}^{\eta}$ is deterministic. Define its TF-domain block average as

$$\bar{\gamma}_{\mathrm{b}}^{\eta} \triangleq \frac{1}{MN} \sum_{m=0}^{M-1} \sum_{n=0}^{N-1} \gamma_{m,n}^{\eta}, \quad \text{(S4)}$$

and decompose

$$\gamma_{m,n}^{\eta} = \bar{\gamma}_{\mathrm{b}}^{\eta} + \delta_{m,n}^{\eta}, \qquad \sum_{m=0}^{M-1} \sum_{n=0}^{N-1} \delta_{m,n}^{\eta} = 0. \quad \text{(S5)}$$

Substituting (S5) into (S3), the constant component contributes

$$\frac{\bar{\gamma}_{\mathrm{b}}^{\eta}}{\sqrt{MN}} \left(\sum_{m=0}^{M-1} e^{j2\pi m \Delta l / M} \right) \left(\sum_{n=0}^{N-1} e^{-j2\pi n \Delta k / N} \right), \quad \text{(S6)}$$

which vanishes for $(\Delta l, \Delta k) \neq (0,0)$ by the orthogonality of the DD-domain phase rotations. Hence, for off-mainlobe offsets, the response is determined only by the TF-domain fluctuation component:

$$\left[\widetilde{\mathbf{\Lambda}}_{\eta}\right]_{\Delta l, \Delta k} = \frac{1}{\sqrt{MN}} \sum_{m=0}^{M-1} \sum_{n=0}^{N-1} \delta_{m,n}^{\eta} \chi_{m,n}(\Delta l, \Delta k). \quad \text{(S7)}$$

We now regard the relative DD offset $(\Delta l, \Delta k)$ as a discrete random variable uniformly distributed over the off-mainlobe grid. For a fixed transmitted block, the expectation over $(\Delta l, \Delta k)$ is the average over all off-mainlobe DD samples. Substituting (S7) gives

$$\mathbb{E}_{\Delta l, \Delta k}\left\{ \left[\widetilde{\mathbf{\Lambda}}_{\eta}\right]_{\Delta l, \Delta k} \right\} = \tfrac{1}{(MN-1)\sqrt{MN}} \sum_{m=0}^{M-1} \sum_{n=0}^{N-1} \delta_{m,n}^{\eta} \sum_{(\Delta l, \Delta k) \neq (0,0)} \chi_{m,n}(\Delta l, \Delta k). \quad \text{(S8)}$$

The inner off-mainlobe phase sum satisfies

$$\sum_{(\Delta l, \Delta k) \neq (0,0)} \chi_{m,n}(\Delta l, \Delta k) = \begin{cases} MN - 1, & m = 0,\ n = 0, \\ -1, & \text{otherwise}. \end{cases} \quad \text{(S9)}$$

Using (S9) and $\sum_{m,n} \delta_{m,n}^{\eta} = 0$, we obtain

$$\begin{aligned} &\mathbb{E}_{\Delta l, \Delta k}\left\{ \left[\widetilde{\mathbf{\Lambda}}_{\eta}\right]_{\Delta l, \Delta k} \right\} \\ &= \frac{1}{(MN-1)\sqrt{MN}} \left[MN\delta_{0,0}^{\eta} - \sum_{m=0}^{M-1} \sum_{n=0}^{N-1} \delta_{m,n}^{\eta} \right] \\ &= \frac{\sqrt{MN}}{MN-1} \delta_{0,0}^{\eta}. \end{aligned} \quad \text{(S10)}$$

Thus, under bounded single-bin TF-domain fluctuations,

$$\mathbb{E}_{\Delta l, \Delta k}\left\{ \left[\widetilde{\mathbf{\Lambda}}_{\eta}\right]_{\Delta l, \Delta k} \right\} = \mathcal{O}\Big((MN)^{-1/2} \Big). \quad \text{(S11)}$$

Therefore, for a sufficiently large TF grid, the mean of the randomly sampled complex off-mainlobe response over uniformly distributed relative DD offsets becomes negligible. This is a fixed-block, large-grid approximate zero-mean property; it is not an exact zero-mean statement for every finite transmitted block. In the special case where $\delta_{m,n}^{\eta} = 0$, i.e., $\gamma_{m,n}^{\eta}$ is constant over the TF grid, all integer-grid off-mainlobe responses vanish.

B. Variance and ISL-Based Interference Power

For a fixed transmitted block, $\widetilde{\mathbf{\Lambda}}_\eta$ is deterministic over the RD grid. The randomness of the sidelobe interference comes from the random relative DD locations of the interfering targets, which sample the off-mainlobe entries of $\widetilde{\mathbf{\Lambda}}_\eta$.

The ISL defined in (40) and (52) represents the normalized sidelobe energy of the corresponding RD response. In the unified notation, it is written as

$$\mathrm{ISL}_\eta = \frac{1}{MN} \sum_{(\Delta l, \Delta k) \neq (0,0)} \left| \left[\widetilde{\mathbf{\Lambda}}_\eta \right]_{\Delta l, \Delta k} \right|^2. \tag{S12}$$

When the relative DD offset $(\Delta l, \Delta k)$ is uniformly sampled over the off-mainlobe grid, the average sidelobe-response power is

$$\sigma^2_{\Lambda,q,p,\eta} \triangleq \mathbb{E}_{\Delta l, \Delta k} \left\{ \left| \left[\widetilde{\mathbf{\Lambda}}_\eta \right]_{\Delta l, \Delta k} \right|^2 \right\} = \frac{MN}{MN-1} \mathrm{ISL}_\eta \simeq \mathrm{ISL}_\eta, \tag{S13}$$

where the last approximation is accurate for large MN. Since the off-mainlobe mean in (S10) is negligible in the large-grid sense, $\sigma^2_{\Lambda,q,p,\eta}$ characterizes the sidelobe-response variance.

For the interference from target $q \neq p$ to the tested cell of target p, the sampled sidelobe response is taken at the relative DD offset $(l^\eta_{s,p} - l^\eta_{s,q}, k^\eta_{s,p} - k^\eta_{s,q})$. For a sufficiently long TF sequence, (S7) is a normalized sum of many phase-rotated TF-domain fluctuation terms. If no individual TF-domain fluctuation dominates, a central-limit-type argument gives

$$\left[\widetilde{\mathbf{\Lambda}}_\eta \right]_{l^\eta_{s,p} - l^\eta_{s,q}, k^\eta_{s,p} - k^\eta_{s,q}} \dot{\sim} \mathcal{CN}\left(0, \sigma^2_{\Lambda,q,p,\eta}\right), \tag{S14}$$

where the zero mean is understood in the large-grid approximate sense discussed above.

The corresponding sidelobe-interference contribution is

$$I_{q,p,\eta} = \frac{\alpha^\eta_{s,q}}{\sqrt{MN}} \left[\widetilde{\mathbf{\Lambda}}_\eta \right]_{l^\eta_{s,p} - l^\eta_{s,q}, k^\eta_{s,p} - k^\eta_{s,q}}. \tag{S15}$$

Therefore,

$$\mathbb{E}\{|I_{q,p,\eta}|^2\} = \frac{|\alpha^\eta_{s,q}|^2}{MN} \sigma^2_{\Lambda,q,p,\eta} \simeq \frac{|\alpha^\eta_{s,q}|^2}{MN} \mathrm{ISL}_\eta. \tag{S16}$$

When the relative DD offsets of different interfering targets are independently distributed over sufficiently diverse sidelobe regions, the aggregate sidelobe interference at the target-p cell is

$$\left[\widehat{\mathbf{\Lambda}}^\eta_{\mathbf{I}} \right]_{l^\eta_{s,p}, k^\eta_{s,p}} = \sum_{\substack{q=1 \\ q \neq p}}^{K} I_{q,p,\eta}. \tag{S17}$$

Thus, the aggregate interference can be approximated as

$$\left[\widehat{\mathbf{\Lambda}}^\eta_{\mathbf{I}} \right]_{l^\eta_{s,p}, k^\eta_{s,p}} \dot{\sim} \mathcal{CN}\left(0, P_{i,p,\eta}\right), \tag{S18}$$

where the variance follows by summing the independent single-target contributions:

$$\begin{aligned} P_{i,p,\eta} &\triangleq \sum_{\substack{q=1 \\ q \neq p}}^{K} \mathbb{E}\{|I_{q,p,\eta}|^2\} \\ &= \frac{1}{MN} \sum_{\substack{q=1 \\ q \neq p}}^{K} |\alpha^\eta_{s,q}|^2 \sigma^2_{\Lambda,q,p,\eta} \simeq \frac{\mathrm{ISL}_\eta}{MN} \sum_{\substack{q=1 \\ q \neq p}}^{K} |\alpha^\eta_{s,q}|^2. \end{aligned} \tag{S19}$$

This gives $P_{i,p,\mathrm{BS}}$ by using $\mathrm{ISL}_{\mathrm{BS}}$, and $P_{i,p,\mathrm{E}}$ by using $\mathrm{ISL}_{\mathrm{E}}$.

Finally, combining the desired mainlobe, aggregate sidelobe interference, and filtered noise gives

$$\left[\widehat{\mathbf{\Lambda}}_\eta \right]_{l^\eta_{s,p}, k^\eta_{s,p}} = \mu_{p,\eta} + \left[\widehat{\mathbf{\Lambda}}^\eta_{\mathbf{I}} \right]_{l^\eta_{s,p}, k^\eta_{s,p}} + \left[\widehat{\mathbf{\Lambda}}^\eta_{\mathbf{Z}} \right]_{l^\eta_{s,p}, k^\eta_{s,p}}. \tag{S20}$$

Since the filtered noise is CSCG with variance $P_{n,\eta}$ and is independent of the sidelobe interference, the RD output satisfies

$$\left[\widehat{\mathbf{\Lambda}}_\eta \right]_{l^\eta_{s,p}, k^\eta_{s,p}} \dot{\sim} \mathcal{CN}\left(\mu_{p,\eta}, P_{i,p,\eta} + P_{n,\eta}\right). \tag{S21}$$

This is the effective CSCG model used in the KLD formulation.

C. LMMSE and MF Receiver Filtering

The BS-side result follows by setting $\eta = \mathrm{BS}$ in (S1), which yields the LMMSE weighting

$$\gamma^{\mathrm{BS}}_{m,n} = \frac{|x_{m,n}|^2}{|x_{m,n}|^2 + \sigma^2_{\mathbf{z}_{\mathrm{BS,s}}}}. \tag{S22}$$

The corresponding ISL is $\mathrm{ISL}_{\mathrm{BS}}$ in (40), which determines $P_{i,p,\mathrm{BS}}$ through (S19).

The Eve-side result follows by setting $\eta = \mathrm{E}$, which gives the MF weighting

$$\gamma^{\mathrm{E}}_{m,n} = |x_{m,n}|^2. \tag{S23}$$

The corresponding ISL is $\mathrm{ISL}_{\mathrm{E}}$ in (52), which determines $P_{i,p,\mathrm{E}}$ through (S19).

For a single interfering target, (S7) shows that the sampled sidelobe response is a normalized sum of many phase-rotated TF-domain fluctuation terms. Hence, when the TF sequence is sufficiently long and no dominant TF-domain fluctuation exists, it can be approximated as a zero-mean CSCG variable as in (S14). If a dominant TF-domain component exists, the individual sidelobe sample may deviate from the CSCG approximation.

For multiple interfering targets, the aggregate sidelobe interference is the sum of independently sampled sidelobe contributions with different phase rotations, as shown in (S17). Therefore, by a central-limit argument, it can be directly modeled as a zero-mean CSCG variable as in (S18), with variance determined by the ISL-based power in (S19).

In summary, for a single target without dominant TF-domain fluctuations, the sampled sidelobe response is approximately CSCG; for multiple targets, the aggregate sidelobe interference can also be approximated as CSCG.

Supplementary Note 2. The Approximation for the KLD

A. Jensen-Based Approximation

For notational brevity, we use $\eta \in \{\mathrm{BS}, \mathrm{E}\}$ to denote the receiver and omit the target index p when no ambiguity arises. Let $T_\eta \triangleq \left|\left[\widehat{\mathbf{\Lambda}}_\eta\right]_{l,k}\right|^2$, $\sigma_\eta^2 \triangleq P_{i,p,\eta} + P_{n,\eta}$, and $\mu_\eta \triangleq \mu_{p,\eta}$. Also write $\mathbb{E}_{1,\eta}[\cdot] \triangleq \mathbb{E}_{T_\eta \sim f_{1,\eta}}[\cdot]$, where $f_{1,\eta}$ denotes the distribution of T_η under $\mathcal{H}_{1,\eta}$. From (63) and the corresponding expression for Eve, the KLD between $\mathcal{H}_{1,\eta}$ and $\mathcal{H}_{0,\eta}$ can be written as

$$D_\eta = \mathbb{E}_{1,\eta}\left[\ln I_0\left(\frac{2|\mu_\eta|\sqrt{T_\eta}}{\sigma_\eta^2}\right)\right] - \frac{|\mu_\eta|^2}{\sigma_\eta^2}. \tag{S24}$$

Define $X_\eta \triangleq 2|\mu_\eta|\sqrt{T_\eta}/\sigma_\eta^2$, $g(x) \triangleq \ln I_0(x)$, and $\xi_\eta \triangleq |\mu_\eta|^2/\sigma_\eta^2$. Then $D_\eta = \mathbb{E}_{1,\eta}[g(X_\eta)] - \xi_\eta$. Since $g(x)$ is convex for $x \ge 0$, Jensen's inequality gives $\mathbb{E}_{1,\eta}[g(X_\eta)] \ge g(\mathbb{E}_{1,\eta}[X_\eta])$. Accordingly, the Jensen lower-bound approximation is

$$D_\eta \approx \widetilde{D}_\eta = g(m_\eta) - \xi_\eta, \quad m_\eta \triangleq \mathbb{E}_{1,\eta}[X_\eta]. \tag{S25}$$

Under $\mathcal{H}_{1,\eta}$, the RD-domain output at the tested bin follows $\left[\widehat{\mathbf{\Lambda}}_\eta\right]_{l,k} \tilde{\sim} \mathcal{CN}(\mu_\eta, \sigma_\eta^2)$. Hence, using the Rice-amplitude moment, the mean of X_η is

$$m_\eta = \mathbb{E}_{1,\eta}[X_\eta] = \frac{2|\mu_\eta|}{\sigma_\eta^2}\mathbb{E}_{1,\eta}\left[\sqrt{T_\eta}\right] = \frac{2|\mu_\eta|}{\sigma_\eta}A(\xi_\eta), \tag{S26}$$

where [1]

$$A(\xi) \triangleq \frac{\sqrt{\pi}}{2}\exp\left(-\frac{\xi}{2}\right)\left[(1+\xi)I_0\left(\frac{\xi}{2}\right) + \xi I_1\left(\frac{\xi}{2}\right)\right]. \tag{S27}$$

Here, $I_0(\cdot)$ and $I_1(\cdot)$ denote the zeroth- and first-order modified Bessel functions of the first kind, respectively. Substituting (S26) into (S25) yields

$$\widetilde{D}_\eta = \ln I_0\left(\frac{2|\mu_\eta|}{\sigma_\eta}A(\xi_\eta)\right) - \xi_\eta. \tag{S28}$$

For $\eta = \mathrm{BS}$, substituting (S27) into (S28) gives (65). The Eve-side approximation is obtained by replacing the BS-side parameters with the corresponding Eve-side parameters.

B. Approximation Error Bound

The approximation error is the Jensen gap

$$\epsilon_\eta \triangleq D_\eta - \widetilde{D}_\eta = \mathbb{E}_{1,\eta}\left[g(X_\eta)\right] - g(m_\eta) \ge 0. \tag{S34}$$

Applying Taylor's theorem to $g(X_\eta)$ around m_η gives

$$g(X_\eta) = g(m_\eta) + g'(m_\eta)(X_\eta - m_\eta) + \frac{1}{2}g''(\zeta_\eta)(X_\eta - m_\eta)^2, \tag{S35}$$

where ζ_η lies between X_η and m_η. Taking expectation and using $\mathbb{E}_{1,\eta}[X_\eta - m_\eta] = 0$, we obtain

$$\epsilon_\eta = \frac{1}{2}\mathbb{E}_{1,\eta}\left[g''(\zeta_\eta)\left(X_\eta - m_\eta\right)^2\right]. \tag{S36}$$

Let $r(x) \triangleq I_1(x)/I_0(x)$. Since $I_0'(x) = I_1(x)$, we have $g'(x) = r(x)$. Using the standard derivative identity $I_1'(x) = I_0(x) - I_1(x)/x$, for $x > 0$,

$$g''(x) = r'(x) = 1 - r^2(x) - \frac{r(x)}{x}. \tag{S37}$$

At the origin, (S31) and (S32) give

$$g''(0) = \lim_{x \to 0}\left(1 - r^2(x) - \frac{r(x)}{x}\right) = \frac{1}{2}.$$

In addition, the modified-Bessel ratio satisfies $0 \le r'(x) \le r(x)/x$ for $x > 0$ [3]. Since this also implies that $r(x)/x$ is decreasing, and $r(x) = x/2 + \mathcal{O}(x^3)$ as $x \to 0$, we have

$$0 \le g''(x) = r'(x) \le \frac{r(x)}{x} \le \frac{1}{2}, \quad x \ge 0. \tag{S38}$$

Substituting (S38) into (S36) yields

$$0 \le \epsilon_\eta \le \frac{1}{4}\mathrm{Var}_{1,\eta}(X_\eta). \tag{S39}$$

The variance of X_η follows from its first two moments. From (S26),

$$\mathbb{E}_{1,\eta}[X_\eta] = \frac{2|\mu_\eta|}{\sigma_\eta}A(\xi_\eta).$$

Also,

$$\begin{aligned}\mathbb{E}_{1,\eta}[X_\eta^2] &= \frac{4|\mu_\eta|^2}{\sigma_\eta^4}\mathbb{E}_{1,\eta}[T_\eta] \\ &= \frac{4|\mu_\eta|^2}{\sigma_\eta^4}\left(|\mu_\eta|^2 + \sigma_\eta^2\right) = 4\xi_\eta(1+\xi_\eta).\end{aligned} \tag{S40}$$

Hence,

$$\mathrm{Var}_{1,\eta}(X_\eta) = 4\xi_\eta\left[1 + \xi_\eta - A^2(\xi_\eta)\right], \tag{S41}$$

and the Jensen gap satisfies

$$0 \le \epsilon_\eta \le \xi_\eta\left[1 + \xi_\eta - A^2(\xi_\eta)\right]. \tag{S42}$$

C. Low-effective SNR Asymptotic Behavior

Let $V_\eta = W_\eta/\sigma_\eta \sim \mathcal{CN}(0,1)$. By circular symmetry,

$$X_\eta = 2\sqrt{\xi_\eta}\frac{1}{\sigma_\eta}\left|\mu_\eta + W_\eta\right| = 2\sqrt{\xi_\eta}\left|\sqrt{\xi_\eta} + V_\eta\right|.$$

We first expand the deterministic function $A(\xi)$. Substituting (S30), (S31), and (S32) into (S27) gives

$$\begin{aligned}A(\xi) &= \frac{\sqrt{\pi}}{2}\left(1 - \frac{\xi}{2} + \mathcal{O}(\xi^2)\right)\left(1 + \xi + \mathcal{O}(\xi^2)\right) \\ &= \frac{\sqrt{\pi}}{2}\left(1 + \frac{\xi}{2} + \mathcal{O}(\xi^2)\right).\end{aligned} \tag{S43}$$

Therefore,

$$\begin{aligned}m_\eta &= 2\sqrt{\xi_\eta}A(\xi_\eta) = \sqrt{\pi\xi_\eta}\left(1 + \frac{\xi_\eta}{2} + \mathcal{O}(\xi_\eta^2)\right), \\ m_\eta^2 &= \pi\xi_\eta + \mathcal{O}(\xi_\eta^2).\end{aligned} \tag{S44}$$

Moreover,

$$\mathbb{E}_{1,\eta}[X_\eta^2] = 4\xi_\eta(1+\xi_\eta) = 4\xi_\eta + \mathcal{O}(\xi_\eta^2). \tag{S45}$$

TABLE S1
STANDARD ASYMPTOTIC EXPANSIONS USED IN SUPPLEMENTARY NOTE 2

Function	Limit	Expansion	
$\ln(1+x)$	$x \to 0$	$\ln(1+x) = x + \mathcal{O}(x^2)$.	(S29)
$\exp(-x/2)$	$x \to 0$	$\exp(-x/2) = 1 - \frac{x}{2} + \mathcal{O}(x^2)$.	(S30)
$I_0(x)$	$x \to 0$	$I_0(x) = 1 + \frac{x^2}{4} + \mathcal{O}(x^4)$.	(S31)
$I_1(x)$	$x \to 0$	$I_1(x) = \frac{x}{2} + \mathcal{O}(x^3)$.	(S32)
$I_\nu(z)$ [2]	$z \to \infty$	$I_\nu(z) = \frac{e^z}{\sqrt{2\pi z}}\left[1 - \frac{4\nu^2-1}{8z} + \frac{(4\nu^2-1)(4\nu^2-9)}{2!\,8^2z^2} + \mathcal{O}(z^{-3})\right]$.	(S33)

To analyze the two terms in (S34), we use the small-argument expansion of $g(x) = \ln I_0(x)$. From (S31) and (S29), we have

$$g(x) = \frac{x^2}{4} + \mathcal{O}(x^4), \quad x \to 0. \tag{S46}$$

Since $m_\eta = \mathcal{O}(\sqrt{\xi_\eta})$, it follows that $m_\eta^4 = \mathcal{O}(\xi_\eta^2)$, and hence

$$g(m_\eta) = \frac{m_\eta^2}{4} + \mathcal{O}(\xi_\eta^2). \tag{S47}$$

For the expectation term, the same expansion is controlled by the deterministic remainder bound $\left|g(x) - x^2/4\right| \le Cx^4$ for $x \ge 0$ as (S46). Thus,

$$\begin{aligned}\left|\mathbb{E}_{1,\eta}[g(X_\eta)] - \frac{1}{4}\mathbb{E}_{1,\eta}[X_\eta^2]\right| &= \left|\mathbb{E}_{1,\eta}\left[g(X_\eta) - \frac{X_\eta^2}{4}\right]\right| \\ &\le \mathbb{E}_{1,\eta}\left[\left|g(X_\eta) - \frac{X_\eta^2}{4}\right|\right] \\ &\le C\mathbb{E}_{1,\eta}[X_\eta^4]. \end{aligned}\tag{S48}$$

Moreover,

$$X_\eta^4 = 16\xi_\eta^2 \left|\sqrt{\xi_\eta} + V_\eta\right|^4.$$

Since $V_\eta \sim \mathcal{CN}(0,1)$ has finite fourth-order moment, $\mathbb{E}_{1,\eta}[X_\eta^4] = \mathcal{O}(\xi_\eta^2)$. Therefore,

$$\mathbb{E}_{1,\eta}[g(X_\eta)] = \frac{1}{4}\mathbb{E}_{1,\eta}[X_\eta^2] + \mathcal{O}(\xi_\eta^2). \tag{S49}$$

Thus,

$$\begin{aligned}\epsilon_\eta &= \mathbb{E}_{1,\eta}[g(X_\eta)] - g(m_\eta) \\ &= \frac{1}{4}\left(\mathbb{E}_{1,\eta}[X_\eta^2] - m_\eta^2\right) + \mathcal{O}(\xi_\eta^2) \\ &= \left(1 - \frac{\pi}{4}\right)\xi_\eta + \mathcal{O}(\xi_\eta^2), \quad \xi_\eta \to 0. \end{aligned}\tag{S50}$$

Therefore, $\epsilon_\eta = \mathcal{O}(\xi_\eta)$ in the low-effective-SNR regime.

D. High-effective SNR Asymptotic Behavior

We next consider the high-effective-SNR regime, i.e., $\xi_\eta \to \infty$. Applying (S33) with $z = \xi/2$ and $\nu = 0, 1$, respectively, gives

$$\begin{aligned} e^{-\xi/2} I_0(\xi/2) &= \frac{1}{\sqrt{\pi\xi}}\left[1 + \frac{1}{4\xi} + \frac{9}{32\xi^2} + \mathcal{O}(\xi^{-3})\right], \\ e^{-\xi/2} I_1(\xi/2) &= \frac{1}{\sqrt{\pi\xi}}\left[1 - \frac{3}{4\xi} - \frac{15}{32\xi^2} + \mathcal{O}(\xi^{-3})\right]. \end{aligned}\tag{S51}$$

Substituting these expansions into $A(\xi)$, we obtain

$$\begin{aligned} A(\xi) &= \frac{1}{2\sqrt{\xi}}\Bigg[(1+\xi)\left(1 + \frac{1}{4\xi} + \frac{9}{32\xi^2} + \mathcal{O}(\xi^{-3})\right) \\ &\qquad + \xi\left(1 - \frac{3}{4\xi} - \frac{15}{32\xi^2} + \mathcal{O}(\xi^{-3})\right)\Bigg] \\ &= \frac{1}{2\sqrt{\xi}}\left[2\xi + \frac{1}{2} + \frac{1}{16\xi} + \mathcal{O}(\xi^{-2})\right] \\ &= \sqrt{\xi} + \frac{1}{4\sqrt{\xi}} + \frac{1}{32\xi^{3/2}} + \mathcal{O}(\xi^{-5/2}). \end{aligned}\tag{S52}$$

Hence,

$$\begin{aligned} m_\eta &= 2\sqrt{\xi_\eta}A(\xi_\eta) = 2\xi_\eta + \frac{1}{2} + \frac{1}{16\xi_\eta} + \mathcal{O}(\xi_\eta^{-2}), \\ m_\eta^2 &= 4\xi_\eta^2 + 2\xi_\eta + \frac{1}{2} + \mathcal{O}(\xi_\eta^{-1}). \end{aligned}\tag{S53}$$

Since

$$\mathbb{E}_{1,\eta}[X_\eta^2] = 4\xi_\eta(1+\xi_\eta),$$

we have

$$\mathrm{Var}_{1,\eta}(X_\eta) = 2\xi_\eta + \mathcal{O}(1). \tag{S54}$$

We now use the Taylor representation in (S36). Recall that by circular symmetry,

$$X_\eta = 2\sqrt{\xi_\eta}\left|\sqrt{\xi_\eta} + V_\eta\right|.$$

Since V_η has finite moments of any order, X_η is concentrated around its leading term in the high-SNR sense, i.e.,

$$X_\eta = 2\xi_\eta + \mathcal{O}(\sqrt{\xi_\eta}).$$

Together with $m_\eta = 2\xi_\eta + \mathcal{O}(1)$, the intermediate point ζ_η between X_η and m_η is of order ξ_η in the high-SNR regime. Furthermore, applying (S33) to $I_0(x)$ gives

$$g(x) = \ln I_0(x) = x - \frac{1}{2}\ln(2\pi x) + \frac{1}{8x} + \mathcal{O}(x^{-2}), \quad x \to \infty, \tag{S55}$$

and therefore

$$g''(x) = \frac{1}{2x^2} + \mathcal{O}(x^{-3}). \tag{S56}$$

Thus, $g''(\zeta_\eta) = \mathcal{O}(\xi_\eta^{-2})$, and from (S36),

$$\begin{aligned}\epsilon_\eta &= \frac{1}{2}\mathbb{E}_{1,\eta}\left[g''(\zeta_\eta)(X_\eta - m_\eta)^2\right] \\ &\le \mathcal{O}(\xi_\eta^{-2})\mathbb{E}_{1,\eta}\left[(X_\eta - m_\eta)^2\right] \\ &= \mathcal{O}(\xi_\eta^{-2})\,\mathrm{Var}_{1,\eta}(X_\eta) \\ &= \mathcal{O}(\xi_\eta^{-1}), \quad \xi_\eta \to \infty. \end{aligned} \tag{S57}$$

Thus, the Jensen gap vanishes in the high-effective-SNR regime.

REFERENCES

[1] S. Ghosal and R. Maitra, “The envelope of a complex Gaussian random variable,” *arXiv preprint arXiv:2305.03038*, 2025.

[2] “NIST digital library of mathematical functions,” https://dlmf.nist.gov/, Release 1.2.6 of 2026-03-15, f. W. J. Olver, A. B. Olde Daalhuis, D. W. Lozier, B. I. Schneider, R. F. Boisvert, C. W. Clark, B. R. Miller, B. V. Saunders, H. S. Cohl, and M. A. McClain, eds.

[3] D. E. Amos, “Computation of modified Bessel functions and their ratios,” *Math. Comput.*, vol. 28, no. 125, pp. 239–251, 1974.
